# Cognitive-behavioral treatment with behavioral activation for smoking cessation: Randomized controlled trial

**DOI:** 10.1371/journal.pone.0214252

**Published:** 2019-04-08

**Authors:** Carmela Martínez-Vispo, Rubén Rodríguez-Cano, Ana López-Durán, Carmen Senra, Elena Fernández del Río, Elisardo Becoña

**Affiliations:** 1 Smoking Cessation and Addictive Disorders Unit, Department of Clinical Psychology and Psychobiology, Faculty of Psychology, University of Santiago de Compostela, Galicia, Spain; 2 Department of Clinical Psychology and Psychobiology, Faculty of Psychology, University of Santiago de Compostela, Galicia, Spain; 3 Department of Psychology and Sociology, University of Zaragoza, Zaragoza, Spain; Brown University, UNITED STATES

## Abstract

**Introduction:**

Behavioral Activation is a behavioral-based treatment that has been proposed as suitable for smoking cessation, as it simultaneously addresses reinforcement-related variables and also mood management. The aim of this study was to compare the effects of a cognitive-behavioral smoking cessation treatment with components of behavioral activation (SCBSCT-BA) with a standard cognitive-behavioral treatment (SCBSCT), and a wait-list control group (WL).

**Method:**

The sample was comprised of 275 adults smokers (61.4% females, mean age = 45.36, *SD* = 10.96). After baseline assessment sessions, participants were randomized (ratio: 2.2.1.) to SCBSCT-BA, SCBSCT, or WL. Active groups received 8 weekly 1-hour face-to-face group sessions. Biochemically verified smoking abstinence and depressive symptoms were assessed at the end of treatment, and at 3-, 6-, and 12-month follow-ups.

**Results:**

Significant treatment effects in 7-dayspoint prevalence abstinence rates were found for both active groups at the end of treatment. Abstinence rates at 12-months follow-up were 30% for SCBSCT-BA, and 18% for SCBSCT. Using Multiple Imputation for missing data, regression analysis showed significantly greater ORs for the SCBSCT-BA condition (vs. SCBSCT) at the end of treatment and at 3-months follow-up. At 6-, and 12-months follow-ups, ORs for the SCBSCT-BA condition, although greater, did not reach statistical significance. Multilevel analysis showed that abstinence was related to reductions in depressive symptoms.

**Conclusions:**

SCBSCT-BA obtained positive results at short and medium term. Participants who quit smoking experienced a significant reduction in depressive symptoms. Findings support the benefit of adding BA to a cognitive-behavioral smoking cessation treatment.

**Trial registration:**

www.clinicaltrials.gov NCT02844595.

## Introduction

Smoking cessation treatments have shown to significantly aid in the achievement of tobacco abstinence [1]. Despite the evidence of the efficacy of such treatments to quit smoking, in recent years, a reduction in abstinence rates has been observed [2–4]. Previous work suggests that this phenomenon could be due to a change in treatment-seeking smokers. For example, smokers of recent cohorts show significantly greater self-reported depressive symptoms when compared to prior studies [2,4]. Research had consistently shown that depressive symptoms and negative affect are highly associated with smoking behavior [5], and with difficulties in quit smoking and maintain abstinence [6–8].

Quitting smoking is a complex process that involves psychological, physiological, and environmental variables. Reinforcement processes, for example, are relevant factors implicated in substance use onset and maintenance [9]. In the case of cigarette smoking, positive reinforcement acts through nicotine and non-nicotine effects, providing pleasure by smoking itself and/or enhancing the reward value of activities or stimuli associated with smoking [10]. Another important mechanism implied in smoking maintenance is negative reinforcement. This process assumes the use of smoking as a way of avoiding nicotine withdrawal symptoms, and also as a way to manage negative affect or as a strategy to avoid and/or escape from psychological distress in general [11]. Moreover, behavioral approaches have suggested that a low natural positive reinforcement in the environment is implicated in the onset and maintenance of smoking behavior, through the lack of rewarding smoking-free stimuli and/or activities [12]. Lastly, studies have shown that the interest, pleasure, and enjoyment of nonsmoking-related activities can be reduced during the smoking cessation process [13].

Because in depression a diminished exposure to positive rewarding activities or the presence of dysfunctional patterns of behavioral avoidance is also common [14,15], reinforcement-related factors would be especially relevant for smokers with depressive symptoms. For instance, previous research has pointed out that smokers with depressive symptoms perceive more benefits and reward value of cigarettes than of alternative rewards when compared to non-depressed smokers [16], and depression-prone smokers have strong expectations and beliefs about the reduction of negative affect and the increase of positive affect through smoking [17]. These variables also have an impact on smoking cessation outcomes. Several studies indicate that depression-prone smokers experience an increase in negative affect and a reduction in positive affect when they try to quit smoking, leading to a higher probability of cessation failure or relapse [18].

Due to the prevalence of depressive symptoms is clearly higher among smokers than among those who have never smoked or quit smoking [19], development of smoking cessation treatments that take into account such symptomatology is needed. In fact, previous research has found that specific treatments adding behavioral mood management components to standard smoking cessation treatments increase abstinence rates [8]. Nevertheless, a recent systematic review assessing the effectiveness of smoking cessation treatments in smokers with major depression or depressive symptoms found that the studies conducted to date are insufficient to establish a clear conclusion about the effectiveness of such treatments [20]. Consequently, the authors highlight the need of further research addressing issues of the previous studies, such as, sample sizes, heterogeneity of targeted populations, or the presence of other comorbid conditions. In addition, while there exist numerous studies examining the efficacy of pharmacological (nicotine replacement therapy, bupropion or varenicline) and combined (pharmacological + behavior therapy or counseling) interventions, research into exclusively cognitive-behavior interventions to quit smoking taking into account depressive symptoms, is still scarce [21].

Literature examining the implication of reinforcement factors on smoking and the relation with depressive symptoms has led to an interest in the development and assessment of smoking cessation treatments addressing reinforcement-related variables, negative mood management, and positive affect promotion by increasing access to rewarding stimuli alternative to cigarettes, and engagement in nonsmoking-related reinforcing and pleasant activities [22]. Behavioral Activation (BA) is a treatment that includes components based on this behavioral approach. This approach could be suitable for smoking cessation, as it simultaneously addresses depressive symptoms and the above-mentioned reinforcement-related variables. BA can be defined as a behavioral-based treatment that follows the principles of operant conditioning in order to promote rewarding experiences, reducing behavioral avoidance patterns and increasing engagement in positively reinforced activities [23].

BA has shown its efficacy and cost-effectiveness, resulting in a front-line treatment for depression [24,25]. In addition, it is also an empirically supported treatment to increase well-being in nonclinical populations [26], and it has obtained positive results when it was included in the treatment of substance use disorders [27–29].

Although BA integrated in smoking cessation treatment has shown preliminary efficacy for improving smoking cessation outcomes and depressive symptoms [30–32], some methodological issues such as sample size or short-term follow-ups prevent clearly confirming its effectiveness. In order to overcome this gap, the current study was designed to evaluate the potential benefit of adding BA components to a cognitive-behavioral smoking cessation treatment. Additionally, most studies address smoking cessation in samples including participants with or without depression separately. As research has confirmed that even very low levels of depressive symptomatology can impact smoking cessation outcomes [33], we did not exclude participants on the basis of their depressive symptoms.

To our knowledge, this study is the largest randomized clinical trial in terms of sample size to evaluate the long-term effects of a BA-based treatment for smoking cessation. This trial was designed to compare a Standard Cognitive-Behavioral Smoking Cessation Treatment plus BA (SCBSCT-BA) to two control conditions, a Standard Cognitive-Behavioral Smoking Cessation Treatment (SCBSCT) condition that matched SCBSCT-BA in treatment contact time, and a Wait-List (WL) condition. This three-arm design allows comparisons between both active groups in order to determine the differential effect of BA, and also comparisons of both active groups to the WL condition, which can serve as a benchmark for assessing the benefits of treatment conditions, and it can provide control over the effects of repeated assessment and the expectancy of receiving treatment.

Therefore, our main objective was to assess the effects of SCBSCT-BA, in terms of smoking abstinence rates and improvement of depressive symptoms, at the end of treatment, and at 3-, 6-, and 12-month follow-ups. Our hypotheses were as follows: (a) individuals randomized to the two active conditions (SCBSCT-BA and SCBSCT) would be more likely to achieve abstinence and to reduce depressive symptoms at the end of treatment than those randomized to the WL condition; (b) individuals randomized to SCBSCT-BA would be more likely to achieve and maintain abstinence at the end of treatment, and at the 3-, 6-, and 12-month follow-ups (vs. SCBSCT) and; (c) individuals randomized to SCBSCT-BA would be more likely to reduce their depressive symptoms at the end of treatment, and at the 3-, 6-, and 12-month follow-ups (vs. SCBSCT).

## Materials and methods

### Setting

This study was conducted at the Smoking Cessation and Addictive Disorders Unit of the University of Santiago de Compostela (Spain) between January 2016 and April 2018. The Bioethics Committee of the University of Santiago de Compostela approved the study, which is registered with the international standard randomized controlled trial number NCT02844595 (www.clinicaltrials.gov).

### Study design and randomization

A three-arm, single blind, randomized controlled design was used to assess the efficacy of a cognitive-behavioral treatment with BA components to quit smoking. Randomization was conducted according to a computer-generated allocation sequence (ratio: 2.2.1.) to the experimental group (SCBSCT-BA), active comparator group (SCBSCT), or wait-list control group (WL). Randomization was not stratified, but it was conducted through blocks of 5 and 10 participants in order to balance enrollment across conditions during the recruitment period.

Researchers conducting assessments were blind to group allocation, which occurred subsequently. Due to the nature of the study, participants were aware that they were assigned to one of the three arms. Nevertheless, participants were not informed about the details of treatment components, and they did not know the research question or the study objectives. After allocation, both active treatments were administered in eight weekly 60-minute sessions. Sessions were performed in groups of 6–8 participants. Post-treatment assessments were carried out during Session 8 (end of treatment), and after these, face-to-face follow-ups were conducted at 3, 6, and 12 months.

The software used for sample size calculation was G*Power3 Software [34]. The sample size was calculated to detect a 20% difference between the two active groups in the proportion of individuals with biochemically confirmed abstinence at the end of treatment (80% power at a two-tailed alpha of .05). A minimum of 102 participants per active group was required. The sample size of the WL control group was set for a minimum of 51 participants. This unbalanced design was chosen because larger abstinence rates were expected in both active groups as compared to the WL.

### Participant recruitment

Participants were recruited through the media, posters in healthcare centers, word of mouth, or referred to the unit by primary care physicians or other specialized services of the public healthcare system. Before participants were enrolled in the study, written informed consent for participation was obtained. No economic compensation for participating in the study was provided.

**Fig 1:**
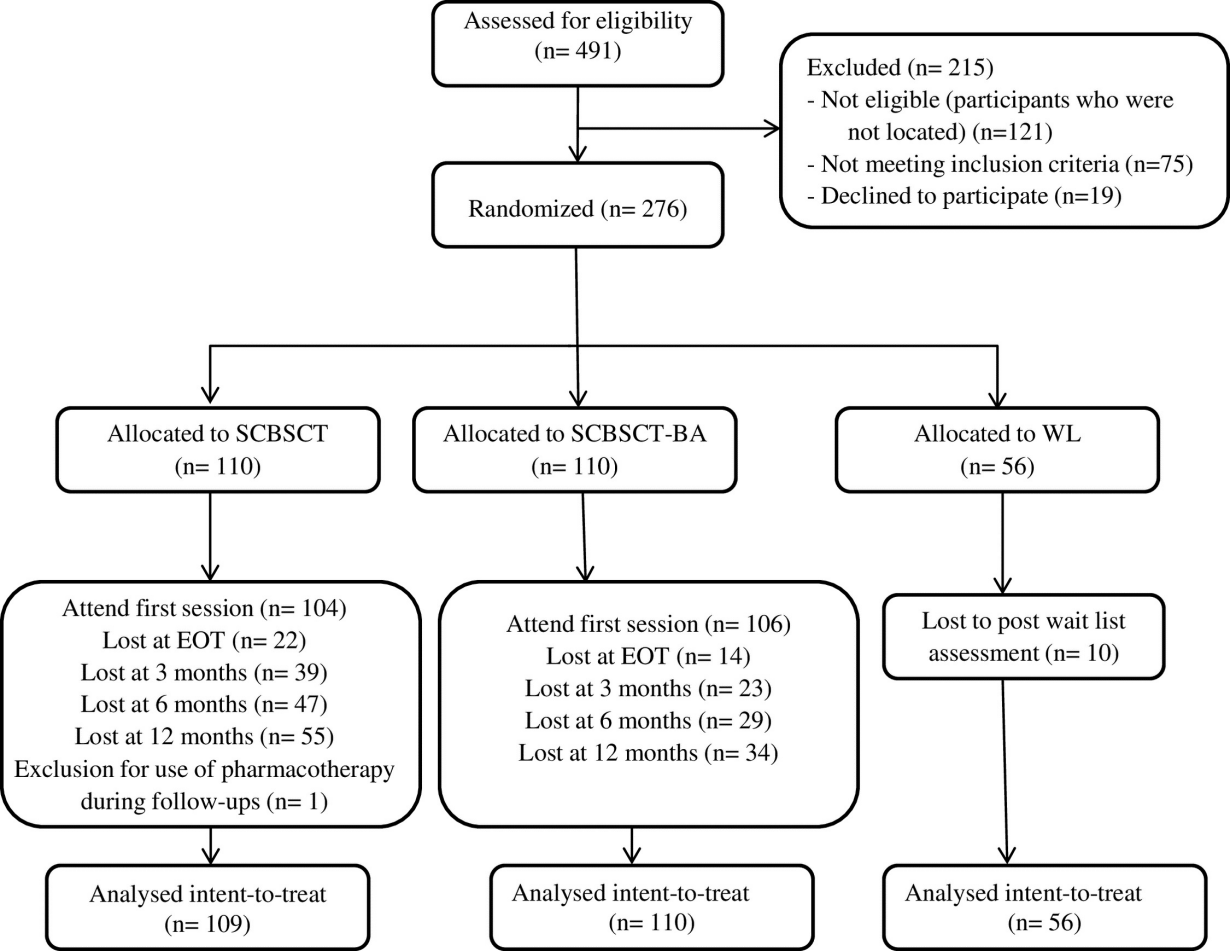
Consort flow chart. Consolidated standards of reporting trials diagram for patient allocation. The randomization ratio was 2.2.1.

Sample selection was carried out according to the following inclusion criteria: aged 18 or over; wishing to participate in the treatment program; providing written informed consent; and smoking at least 8 cigarettes per day. Exclusion criteria were: a diagnosis of severe mental disorder (bipolar disorder and/or psychotic disorder); concurrent substance use disorder (alcohol, cannabis, stimulant, hallucinogen and/or opioid); having participated in the same or similar treatment during the previous year or having received pharmacological smoking cessation treatment (nicotine replacement therapy, bupropion, or varenicline) during the previous year; presence of a high life-risk pathology that would require immediate individual treatment (i.e., recent myocardial infarction); and smoking tobacco products other than cigarettes (i.e., cigars, electronic devices). The overall participant’s flow chart is reported in Fig 1.

### Assessment

Two baseline assessment sessions were carried out. During the first assessment session, sociodemographic characteristics, smoking-related and depression-related variables were collected through a face-to-face structured interview. We also applied the following instruments:

#### Smoking Habit Questionnaire [35]

It consists of 56 items designed to gather information both on sociodemographic variables (sex, age, marital status, educational level) and tobacco use.

#### Fagerström Test for Cigarette Dependence (FTCD) [36–38]

It is made up of six items for the assessment of cigarette dependence. The Spanish version has a Cronbach alpha of .65.

#### Beck Depression Inventory II (BDI-II) [39,40]

This is a 21-item self-report scale measuring current depressive symptoms. The Spanish version has a Cronbach alpha of .90.

#### Environmental Reward Observation Scale (EROS) [41,42]

This is a 10-item self-report measure examining the amount and availability of environmental reward. The Spanish version has a Cronbach’s alpha of .86.

#### Behavioral Activation for Depression Scale (BADS) [43,44]

This 25-item questionnaire was designed to measure the degree to which a person engages in activities. The Spanish version has a Cronbach’s alpha of .90.

#### Client Satisfaction Questionnaire (CSQ-8) [45,46]

This 8-item instrument measure general satisfaction with treatment services. The general score is used organized according to 3 levels of satisfaction: 1) low (8–20); 2) medium (21–26); and 3) high (27–32).

#### Assessment of carbon monoxide (CO) in expired air

This measure was conducted through the use of the Micro+ Smokerlyzer (Bedfont Scientific Ltd, Maidstone, Kent, UK). CO was assessed at baseline, at the end of treatment, and at the 3-, 6-, and 12-month follow-ups to corroborate self-reported abstinence.

#### End of treatment and follow-up questionnaires

During last intervention session (session 8), participants filled in an End of Treatment (EOT) questionnaire [47]. After that, face-to-face follow-ups at 3, 6, and 12 months post-treatment were carried out, at which time participants filled in the corresponding questionnaire.

### Conditions

A full description of the protocol and session-by-session treatment components has been published [48]. Treatment conditions were exclusively cognitive-behavioral-based interventions, and pharmacotherapy was not used. Both active conditions consisted of 8 weekly 1-hour face-to-face group sessions. Quit day was scheduled between sessions 5 and 6 of both active conditions.

#### Standard cognitive-behavioral smoking cessation treatment (SCBST)

It consists of a multicomponent cognitive-behavioral manualized treatment to quit smoking, called “Programa para Dejar de Fumar” (Smoking Cessation Program [49]). Originally, SCBST is applied in 6 sessions, but the number of sessions was extended to 8 in order to allow a time-matched comparator condition. Its components are smoking self-report, information about tobacco, nicotine fading, stimulus control, activities to prevent withdrawal syndrome, physiological feedback on cigarette consumption through CO in expired air, and relapse-prevention strategies (assertion training, problem-solving training, changing tobacco-related misconceptions, management of anxiety and anger, exercise, and weight control).

#### Standard cognitive-behavioral smoking cessation treatment plus BA (SCBST-BA)

BA was applied along with the previously described standard cognitive-behavioral smoking cessation treatment. The amount of time used for the components applied in the standard conditions was readjusted to allow the incorporation of BA procedures. In addition to the mentioned treatment elements, it included: analysis of the relationship between behavior and mood, identification of situations and behaviors that worsen mood, identification of avoidance behaviors and rumination, self-report of pleasant daily activities, and pleasant activity scheduling to increase engagement in non-smoking-related rewarding activities.

#### Wait list (WL)

This was a delayed-treatment control group for a period of 3 months, during which participants did not receive any treatment. Three months after the assessment sessions, another assessment session was carried out, after which participants were offered to participate in a smoking cessation treatment.

### Treatment delivery

Two trained therapists (Master level in Counseling Psychology) with years of experience in the application of the SCBSCT conducted the group treatment sessions. One of the therapists was trained in BA procedures (50 hours supervised by a clinical psychologist) and conducted the CBSCT-BA sessions. In previous studies conducted by our group, therapists’ effect on the application of the SCBSCT was analyzed, finding no significant differences in abstinence rates between therapists[50].

Sessions were video-recorded in order to supervise the study protocol procedures and also adherence to protocol. Supervision was conducted independently by two clinical psychologists, who also provided feedback to the therapists. As mentioned before, both treatments were manualized, including a detailed session-by-session protocol and follow-up procedures.

### Outcomes

#### Primary outcomes

The smoking-related primary outcome was point-prevalence abstinence defined according to Russell Standard (RS) criteria [51]. Participants were considered abstinent if they reported abstinence, not even a puff of a cigarette, for ≥ 7 days at the end of treatment, 3-, 6- and 12-month follow-ups, and had an expired CO reading of ≤ 9 parts per million (ppm) [52]. Participants were considered smokers when they reported to be abstinent but had a CO reading of ≥ 10 ppm, or reported being smokers during the face-to-face follow-up session or by telephone.

The depression-related primary outcome was the longitudinal examination of depressive symptoms, assessed through the BDI-II, in each active group.

#### Secondary outcomes

We examined whether there was a reduction of cigarette consumption over time in those participants smoking at the 12-month follow-up, comparing treatment conditions. We also analyzed the effect size of depressive symptoms change, assessed through the BDI-II, behavioral activation, assessed through BADS, and environmental reward, assessed through EROS, from baseline to the 12-month follow-up, in each condition. Finally, we examined the session’s attendance in each condition and its mediation role in abstinence outcomes.

### Statistical analysis

Chi-square tests and one-way ANOVA tests were used to evaluate differences between CBSCT, CBSCT-BA, and WL groups on demographics, smoking-related and depression-related variables. To account for multiple testing, we applied the Bonferroni-adjusted significance level for the chi-square test. To determine effect sizes, we used Cramer's *V*, Cohen’s *d*, and partial eta-square (*η*_*p*_^2^).

Analyses were conducted using intention-to-treat, which includes in the primary analysis all participants who were randomized. In order to handle missing data about primary outcomes (smoking status), we used Multiple Imputation (MI) [53,54]. We imputed missing data with the expectation-maximization (EM) algorithm, which is in the SPSS Missing Value Analysis module [55]. The EM algorithm allows for estimates of missing data from available data via an iterative maximum likely-hood procedure. Thus, we included in the imputation model for smoking status missing data the following variables: treatment condition, demographics (sex, age, marital status, and educational level); cigarette dependence (FTCD), and baseline depression (assessed through BDI-II). One-hundred-fifty imputed data sets were generated, and pooled results are reported. Then, binary logistic regression analyses were conducted to examine abstinence over time (at the end of treatment, at the 3-, 6-, and 12-month follow-ups). Both unadjusted and adjusted analyses (controlling for age, sex, marital status, educational level, baseline cigarette dependence assessed with the FTCD, baseline depression scores on the BDI-II) were conducted.

In order to examine depression-related primary outcome, analyses were also conducted using intention-to-treat. To handle missing data, we used MI through the EM algorithm of the SPSS Missing Value Analysis module. We included in the imputation model the following variables: treatment condition, demographics (sex, age, marital status, and educational level); cigarette dependence (FTCD), smoking status, and baseline depression (assessed through BDI-II). Then, we conducted a Multilevel Analysis (MLM) [56] to examine whether smoking status was related to depressive symptoms at each time point assessment. In this analysis, we included the treatment condition, cigarette dependence (FTCD), sex, and history of depression treatment.

Secondary-related analyses were conducted with those participants with complete data at each point assessment. To analyze longitudinally cigarette consumption, in terms of mean self-reported cigarettes per day, for those who were smoking at the 12-month follow-up, we a used repeated measures analysis of variance (ANOVA). A 2 (SCBSCT vs. SCBSCT-BA) × 5 (repeated measures factor: number of cigarettes per day at baseline, at post-treatment, and at the 3-, 6-, and 12-month follow-ups) approach was used. We used the Greenhouse–Geisser *F* (*FGG*) to correct for the absence of sphericity [57]. Post hoc analyses were performed with the Bonferroni test.

Mediation analyses were performed with PROCCESS macro for SPSS [58], in order to examine whether the number of sessions attended by participants was a mediator between treatment condition (SCBSCT-BA vs. SCBSCT) and smoking outcomes (abstainer vs. smoker). We conducted four models testing this relationship at the end of treatment, and at 3-, 6-, and 12-months follow-ups. Bias-corrected bootstrapping (with 20,000 resamples) was used for assessing indirect effects [59].

SPSS version 24 was used for statistical analysis. The value of the significance level was set at .05.

## Results

### Baseline participant characteristics

Table 1 shows demographics, smoking-related and depression-related variables at baseline. No significant differences were found between groups. Nearly 60% of the sample smoked more than 20 cigarettes per day, 42.5% were nicotine dependent according to the FTCD (score ≥ 6), and the average baseline CO was 18.6 ppm (*SD* = 8.6). Regarding depression-related characteristics, of the total sample, more than 40% had history of treatment for depression; nearly 18% received current treatment for depression. Baseline scores on the BDI-II ranged from 0 to 53, and the mean score was 10.5 (*SD* = 9.1).

**Table 1:** Baseline sociodemographic, smoking-related variables and depression-related variables by trial condition.

	SCBSCT-BA (*n* = 110)	SCBSCT (*n* = 109)	WL (*n* = 56)
	Mean/*n* (*SD*/%)	Mean/*n* (*SD*/%)	Mean/*n* (*SD*/%)
Age (years)	45.24 (11.2)	44.61 (10.7)	47.07 (10.6)
Gender			
Female	67 (60.9)	69 (63.3)	33 (58.9)
Marital status			
Married/living with a partner	52 (47.3)	59 (54.1)	29 (51.8)
Single	36 (32.7)	32 (29.4)	14 (25.0)
Divorced/separated/widowed	22 (20.0)	18 (16.5)	13 (23.2)
Education			
< HS diploma	19 (17.3)	26 (23.9)	14 (25.0)
HS diploma or GED	42 (38.2)	41 (37.6)	22 (39.3)
College or technical school	49 (44.5)	42 (38.5)	20 (35.7)
Current work situation			
Working (yes)	65 (59.1)	63 (57.8)	34 (60.7)
Cigarettes smoked per day	18.85 (7.3)	19.33 (7.4)	19.03 (7.2)
Nicotine content (mg)	0.75 (0.1)	0.74 (0.1)	0.72 (0.2)
Age began daily smoking	18.03 (3.2)	18.31 (3.7)	18.36 (3.6)
Years smoking	26.75 (11.2)	25.24 (11.6)	27.88 (11.3)
Baseline Carbon Monoxide (ppm)	19.22 (8.7)	18.50 (7.2)	17.66 (7.6)
FTCD	4.59 (2.1)	4.95 (2.2)	4.85 (2.1)
Past Depression Treatment (yes)	44 (40.0)	52 (47.7)	23 (41.1)
Current Depression Treatment (yes)	19 (17.3)	20 (18.3)	10 (17.9)
BDI-II	10.27 (8.4)	10.73 (9.6)	10.86 (9.7)

Abbreviations: SCBST-BA = Standard cognitive-behavioral smoking cessation treatment plus BA; SCBST = Standard cognitive-behavioral smoking cessation treatment; WL = wait list; HS = high school; GED = general education diploma; FTCD = Fagerström Test for Cigarette Dependence; BDI-II; Beck Depression Inventory.

### Retention, compliance, and treatment satisfaction

Of the total randomized participants, four assigned to SCBSCT-BA (3.6%), and five to SCBSCT (4.5%) did not attend the group sessions. A total of 10 out of the 56 participants assigned to the WL condition did not attended the post-assessment session (17.8%). Of those participants who attended at least the first treatment session, 95 out of 106 (89.6%) participants of the SCBSCT-BA attended the end of treatment session (session 8), whereas 79 out of 104 participants of the SCBSCT did so (75.9%).

Regarding session attendance, 80.9% of the participants in SCBSCT-BA completed at least 6 group sessions, while in SCBSCT, 67.9% of the participants did so, χ^2^ = 4.87, *p* = .02. Statistical group differences were observed when examining the average session attendance (*M* = 6.7, *SD* = 2.03 for CBSCT-BA vs. *M* = 5.9, *SD* = 2.41 for CBSCT; *t* = 2.71, *p* = .007).

When examining participant’s satisfaction with smoking cessation treatment, they showed high scores according to CSQ-8, with the 94.7% of the participants in SCBSCT-BA condition, and 87.3% in SCBSCT reporting to be highly satisfied. No significant differences were found between both active groups (χ^2^ = 4.09, *p* = .13).

### Primary smoking outcomes

Biochemically verified 7-days point prevalence abstinence rate for the WL control group was 5.4% after the period of 3-months established. Significant differences were found among active conditions at the End of Treatment and the WL control group (64.7% SCBSCT-BA vs. 5.4% WL, *p =* .001; 45.9% SCBSCT vs. 5.4% WL, *p* = .001). When comparing both active groups, significant differences were also found at the end of treatment (Table 2). Concretely, 71 participants of the SCBSCT-BA condition (64.7%) and 50 participants of the SCBSCT condition (45.9%) reported 7-days point prevalence abstinence. At 3-, 6, and 12 months follow-ups significant differences were also found, with participants randomized to the SCBSCT-BA condition achieving significant higher 7-days point prevalence abstinence rates than those of the SCBSCT condition. We also found that at 3-, 6-, and 12-months follow-ups, the rates of missing data were statistically significant higher for those participants randomized to the SCBSCT condition, comparing to those of the SCBSCT-BA condition. No significant differences were found in the category of smokers among treatment conditions.

**Table 2:** Biochemically confirmed abstinence rates, smoking rates and missing data rates by treatment condition (N = 219).

	Abstinence Rates^a^		Smoking rates		Missing data rates			
	SCBSCT-BA n (%)	SCBSCT n (%)	SCBSCT-BA n (%)	SCBSCT n (%)	SCBSCT-BA n (%)	SCBSCT n (%)	*χ*^*2*^	*Cramer*,*s V*
EOT	71 (64.5)^*^	50 (45.9)^*^	25 (22.7)	37 (33.9)	14 (12.7)	22 (20.2)	7.741	.188
3-month follow-up	42 (38.2)^*^	25 (22.9)^*^	45 (40.9)	45 (41.3)	23 (20.9)^*^	39 (35.8)^*^	8.438	.189
6-month follow-up	33 (30.0)^*^	20 (18.3)^*^	48 (43.6)	42 (38.5)	29 (26.4)^*^	47 (43.1)^*^	7.847	.189
12-month follow-up	33 (30.0)^*^	20 (18.3)^*^	43 (39.1)	34 (31.2)	34 (30.9)^*^	55 (50.5)^*^	9.191	.205

*Abbreviations*: EOT = End of Treatment; SCBST-BA = Standard cognitive-behavioral smoking cessation treatment plus BA; SCBST = Standard cognitive-behavioral smoking cessation treatment

*Note*: *p* value corrected by *Bonferroni* method

^a^ Abstinence rates = biochemically verified 7-days point prevalence abstinence

^*^p ≤ 0.01

We performed MI analysis to handle missing data of the primary outcome (biochemically confirmed 7-days point prevalence abstinence). Then, we run the binary logistic regression analysis (Table 3), using both unadjusted and adjusted analysis. At the end of treatment, both adjusted and unadjusted *OR*s were statistically greater for the SCBSCT-BA condition. At the 3-months follow-up the *OR* was statistically greater for the SCBSCT-BA, but when adjusted by covariates did not reach the significance. At 6-, and 12-month follow-ups both abstinence ORs and AORs were greater but statistically nonsignificant for CBSCT-BA.

**Table 3:** Regression analysis biochemically confirmed by treatment condition using Multiple Imputation.

Abstinence Measures	OR^a^ (95% Cl)	*p*	AOR^b^ (95% Cl)	*p*
	SCBSCT-BA vs. SCBSCT		SCBSCT-BA vs. SCBSCT	
EOT^c^	2.15 (1.1, 4.0)	0.012	2.0 (1.0, 3.8)	0.031
3-month follow-up	1.87 (1.0, 3.3)	0.045	1.71 (0.8, 3.2)	0.081
6-month follow-up	1.46 (0.7, 2.7)	0.273	1.36 (0.7, 2.6)	0.373
12-month follow-up	1.40 (0.7, 2.7)	0.302	1.41 (0.7, 2.4)	0.310

*Note*: Abstinence = biochemically verified 7-days point prevalence abstinence (coded as: smoker = 0, abstinent = 1)

^a^OR = Unadjusted odds ratios

^b^AOR = Adjusted odds ratios based on binary logistic regression analysis (sex, age, marital status, educational level, cigarette dependence assessed by FTCD, baseline depressive symptoms scores measured by BDI-II).

^c^EOT = End of Treatment

### Primary depression outcomes

Regarding depression symptoms, no significant differences were found at baseline between the three groups, *F*(2, 272) = 0.153, *p* = .82. At post-treatment, there was a statistically significant group difference as determined by one-way ANOVA, *F*(2, 217) = 3.224, *p* = .04. A Bonferroni post hoc test revealed that the SCBSCT-BA (*p* = .04) and SCBSCT (*p* = .04) groups reported significantly lower depressive symptoms than the WL group at the post assessment session. No significant differences were found at the end of treatment between the active groups (*t* = .655, *p* = .51).

In order to examine the effect of time-varying abstinence on depressive symptoms, a MLM was conducted with the imputed data. Treatment condition, cigarette dependence, sex, and past depression treatment were included as covariates in the model, as they could be also related to depressive symptoms. Results showed that abstinence (vs. smoking) at each point time assessment was related to a reduction in depressive symptoms (Table 4).

**Table 4:** MLM predicting BDI-II scores at end of treatment, and 3-, 6-, and 12- month follow-ups (n = 210).

Parameter	*B*	S.E.	95% C.I.	*p*
Fixed effects				
Intercept	8.12	1.07	(6.02, 10.22)	0.001
Past depression treatment (vs. none)	3.65	0.87	(1.93, 5.37)	0.001
Male (vs. Female)	0.41	0.89	(-1.32, 2.16)	0.468
Cigarette dependent (vs. non-dependent)	2.54	0.97	(0.49, 4.43)	0.021
SCBSCT-BA (vs. SCBSCT)	-0.03	0.86	(-1.68, 1.69)	0.970
Time-varying Smoking Status				
Abstinent (vs. smoker)	-3.98	0.78	(-2.43, -5.52)	0.001
Variance components				
In time-varying smoking abstinence	1.89	2.87	(0.09, 36.91)	0.509

**Fig 2:**
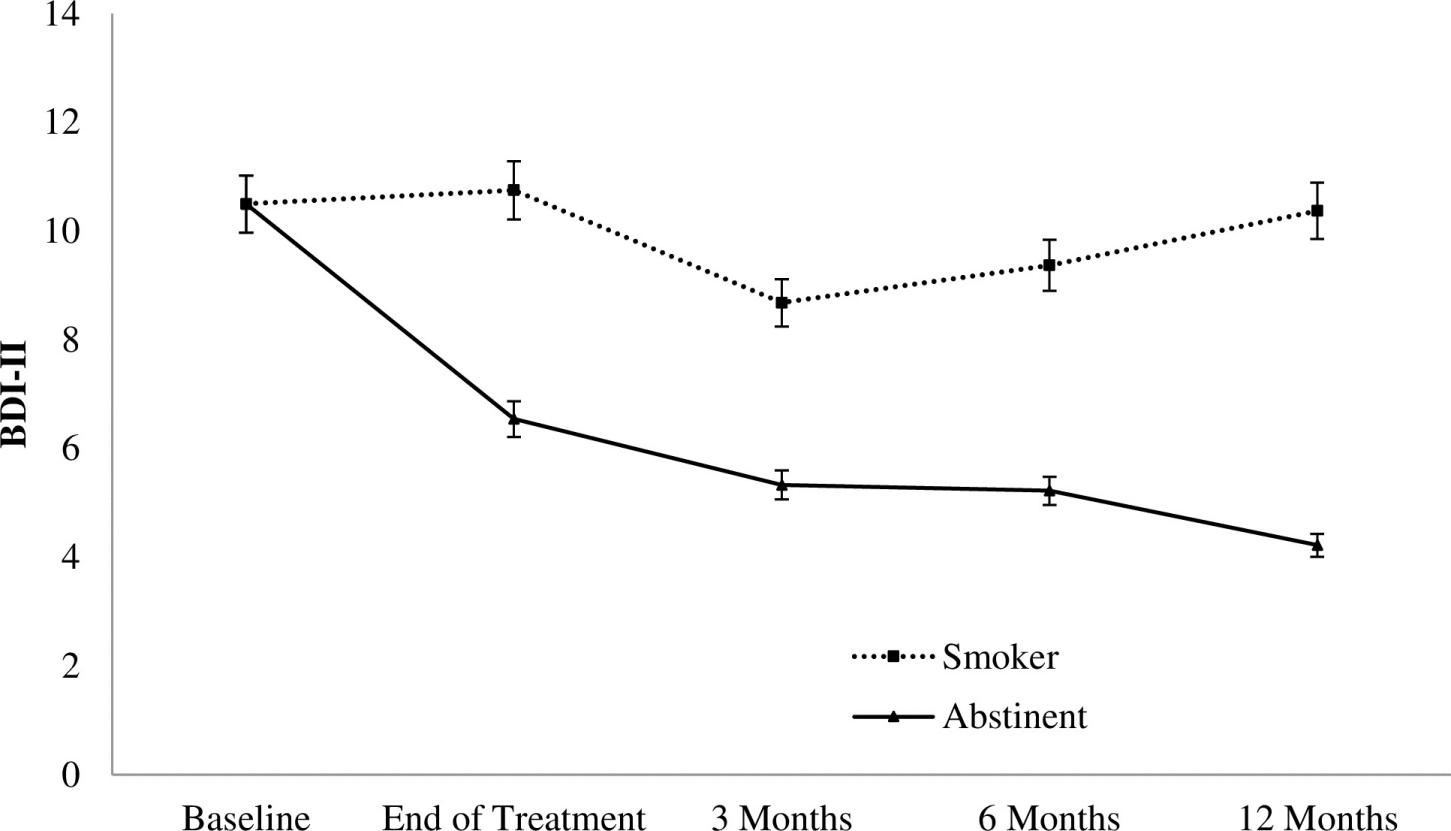
Depressive symptoms by smoking status at the end of treatment, 3-, 6-, and 12-months follow-ups (n = 210). Note. BDI-II: Beck Depression Inventory–Second edition. Included participants were those who attended at least the first treatment session.

Depressive symptoms means assessed through the BDI-II at each point time (baseline, end of treatment, 3-, 6- and 12-months follow-up) by smoking status (smoker vs. abstainer) of those participants with complete data are reported in Fig 2.

### Secondary outcomes

We conducted a repeated-measures ANOVA to examine whether cigarette consumption decreased from baseline to each follow-up point in each treatment condition in the subsample of participants with complete data and that informed to smoke at the 12-month follow-up (*n* = 77). Results showed that participants reduced the number of cigarettes smoked per day across time, *FGG* (3.44, 2962.65) = 106.549, *p* = .001, *η*_*p*_^*2*^ = 0.587. In addition, data showed significant differences between treatment conditions favoring SCBSCT-BA, as participants of this group smoked significantly fewer cigarettes per day compared to SCBSCT, *F*(1, 76) = 7.385, *p =* .008, *η*_*p*_^*2*^ = 0.090).

When examining effect sizes of depressive symptoms reduction from baseline to the 12- month follow up of those participants who provided data in each treatment condition, we found a small to medium effect size for SCBSCT-BA (*d* = 0.39) and a small effect for SCBSCT (*d* = 0.16). In addition, we also examined behavioral activation (BADS) and environmental reward (EROS) change from baseline to end-of treatment (Table 5), finding a higher effect size for both variables in the SCBSCT-BA condition.

**Table 5:** Mean, standard deviations, and effect sizes for depressive symptoms, behavioral activation, and environmental reward from baseline to the end of treatment and at the 3-, 6-, and 12-month follow-ups.

	SCBSCT-BA			SCBSCT		
	Mean	SD	Cohen’s *d*^a^^b^	Mean	SD	Cohen’s *d*^a^^b^
BDI-II						
Pretreatment	10.27	8.41	—	10.73	9.69	—
End of treatment	8.02	6.80	0.29	7.32	7.37	0.37
3-month follow-up	7.78	9.16	0.28	6.77	9.03	0.40
6-month follow-up	6.34	6.08	0.52	9.95	10.23	0.06
12-month follow-up	7.05	7.98	0.39	8.96	10.99	0.16
EROS						
Pretreatment	27.67	4.40	—	27.69	4.93	—
End of treatment	29.50	4.35	0.41	29.11	5.08	0.28
3-month follow-up	29.92	5.14	0.47	28.83	4.65	0.24
6-month follow-up	30.34	4.70	0.58	27.77	5.08	0.02
12-month follow-up	30.48	4.91	0.60	28.55	5.54	0.16
BADS						
Pretreatment	104.58	20.77	—	102.00	25.50	—
End of treatment	108.80	20.92	0.20	106.29	24.50	0.17
3-month follow-up	107.63	24.36	0.13	103.71	24.67	0.07
6-month follow-up	109.14	22.89	0.21	101.77	25.09	0.01
12-month follow-up	110.01	23.65	0.24	102.42	28.43	0.01

*Note*. BDI-II: Beck Depression Inventory–Second edition; EROS = Environmental Reward Observation Scale; BADS = Behavioral Activation Depression Scale

^a^The effect size is the comparison of means at each time point with baseline data.

^b^Each data is based on complete data of participants.

**Fig 3:**
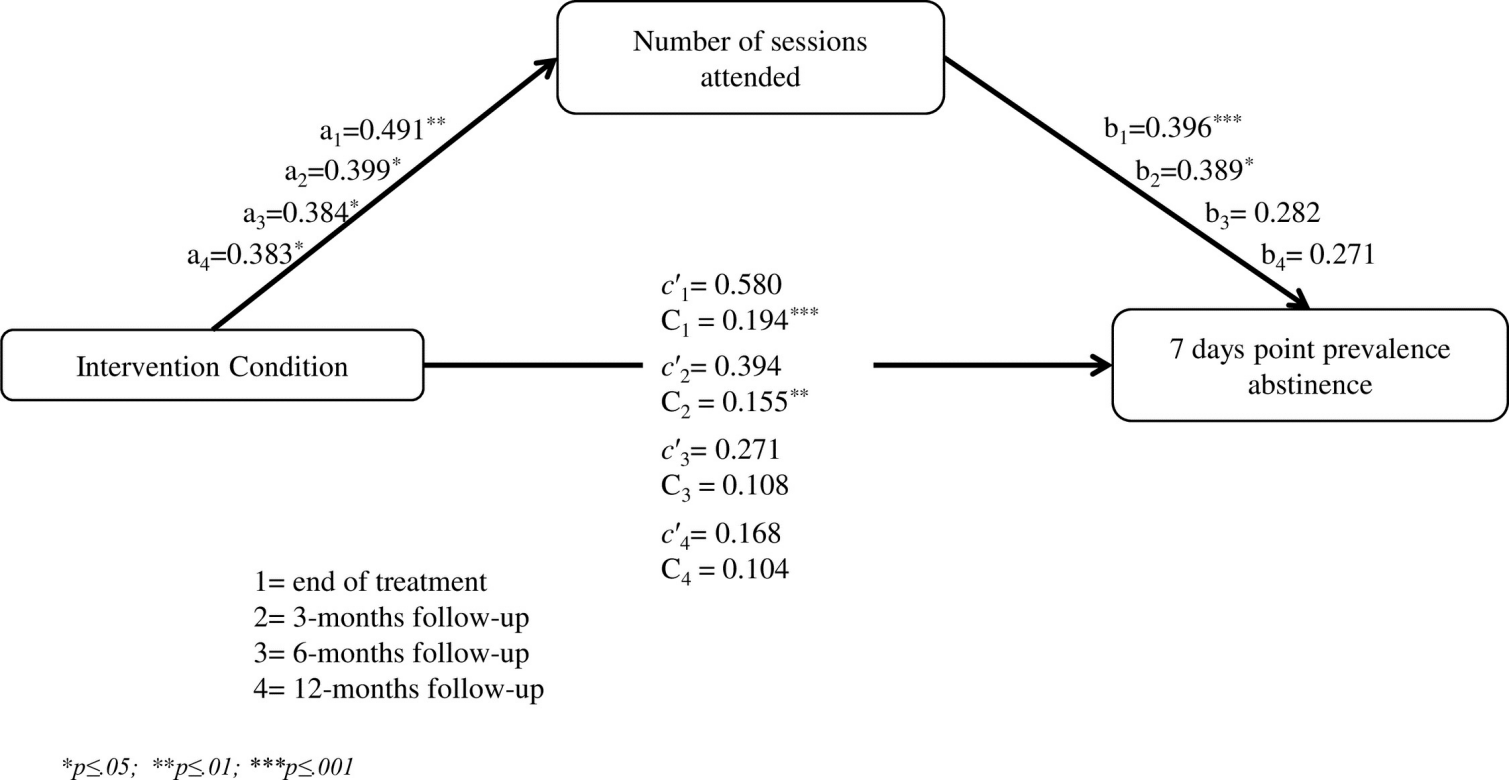
Mediation model for treatment condition, number of sessions attended and 7-days point prevalence abstinence. Direct and indirect effects at the end of treatment, and at 3-, 6-, and 12-months follow-ups.

Due to we found differences in sessions attendance between conditions, we examined whether the number of sessions attended by participants was a mediator between treatment condition (SCBSCT-BA vs. SCBSCT) and smoking 7-days PPA at the end of treatment, and at 3-, 6-, and 12-months follow-ups. These analyses were conducted based on those participants with complete data (Fig 3). Mediation analyses were performed including as covariates gender, age, marital status, education, cigarette dependence (FTCD), and baseline depression (BDI-II). Results of these analysis showed that the indirect effect of the treatment condition through the number of sessions attended by participants was significant at the end of treatment (point estimates = 0.194; SE = 0.117; BC 95% CI [0.031, 0.484]), and at 3-months follow-up (point estimates = 0.155; SE = 0.127; BC 95% CI [0.003, 0.486]), while the direct effect was not significant. The indirect effect did not reach statistically significance at 6-months (point estimates = 0.108; SE = 0.123; BC 95% CI [-0.015, 0.447]), and at 12- months follow-up (point estimates = 0.104; SE = 0.127; BC 95% CI [-0.023, 0.448])

## Discussion

This randomized clinical trial was designed to evaluate the efficacy of SCBSCT-BA compared to SCBSCT, and a WL control group both in smoking cessation and depressive symptom outcomes. Results indicated that there were significant overall differences across the three conditions at the end of treatment. Confirming our first hypothesis, individuals randomized to the two active conditions achieved significantly higher abstinence rates and reported fewer depressive symptoms at the end of treatment than those randomized to the WL condition.

When examining our second hypothesis, we found greater abstinence rates for SCBT-BA when comparing to the active comparator group (SCBSCT) at the end of treatment, and at 3-, 6-, and 12-months follow-up. Nevertheless, when examining regression analysis results, ORs were only statistically significant greater for SCBT-BA condition at the end of treatment and at the 3-month follow-up. Additionally, when analyses were adjusted by covariates, the AORs for SCBT-BA condition were significantly greater only at the end of treatment. Therefore, we only can partially confirm this hypothesis.

Another relevant smoking-related outcome was that the participants who smoked at the 12-month follow-up reported smoking fewer cigarettes per day than at baseline assessment. Although this occurred in both active conditions, SCBSCT-BA participants reported a greater reduction of cigarette consumption when compared to the SCBSCT condition. A plausible explanation is that due to the higher percentage of abstainers in the SCBSCT-BA condition, the rate of participants who relapsed (vs. those who did not stop smoking at the end of treatment) is higher than in the SCBSCT condition. As a result, these participants could have the benefit of the abstinence period, as it has been shown that longer periods of abstinence are associated with greater perceptions of control over smoking behavior, which could translate to reducing the likelihood of re-establishing previous cigarette consumption [60]. Moreover, it is also possible that the participants of the SCBSCT-BA condition would have the benefit of strategies addressing positive rewarding activity alternatives to smoking and, consequently, diminished their cigarette consumption.

Overall, smoking-related results suggest that the BA treatment approach helps smokers to achieve abstinence, and to reduce smoking heaviness (in terms of reduction of the mean number of cigarettes smoked per day) among those who relapsed or continued to smoke after treatment. Thus, the potential benefit of adding BA to a smoking cessation treatment found in previous pilot studies was confirmed [30,31]. As suggested Mckay [61], to address not only aspects directly related to substance use, but also to focus on making abstinence more rewarding, engaging in new hobbies or retaking older ones, making a positive use of leisure time, and performing other activities that grant a sense of meaning to one’s life can make it more likely for participants to reduce their substance use. In addition, due to BA focuses on strategies targeting behaviors in response to negative emotional states and distress [15], participants in the SCBSCT-BA group could benefit from learning coping skills other than smoking when they experience internal negative states.

Regarding our third hypothesis, we expected a greater reduction of depressive symptoms in the SCBSCT-BA group, based on previous research [25,30]. When examining depressive symptoms change from baseline to each follow-up assessment in both treatment conditions, results showed a higher effect size of such change in the SCBSCT-BA condition. Nevertheless, multilevel analysis revealed that treatment condition did not significantly predict depressive symptoms, but instead that time varying-abstinence was related to a reduction in depressive symptomatology at each time point assessment. This finding is in line with previous research that found an association between smoking abstinence and depressive symptom reduction [62–64]. In fact, it has been shown that quitting smoking is associated with a decrease in depressive symptoms, and also with a reduction in anxiety, stress levels, and an improvement in quality of life in people with and without psychiatric disorders [65].

These findings could indicate that the mechanism through which BA increases the likelihood of quitting is not through its impact on depression. In previous literature on mood management interventions for depressed smokers, a similar pattern has been found. Concretely, mood-focused interventions have been shown to increase efficacy in terms of smoking abstinence, but not in reducing depression [66]. A possible explanation of this result could be that participants may have engaged in activities that are incompatible with smoking, or activities not previously associated with tobacco use, which are not necessarily related to depression. In fact, there is evidence showing that increased engagement in substitute reinforcers (alternative activities to smoking), and decreased engagement in complementary reinforcers (activities that increase the reinforcing value of tobacco) are associated with smoking abstinence [67,68]. More research is needed to clarify the mechanism through which BA components impact on smoking outcomes.

Regarding retention data, we found that participants in the SCBSCT-BA group attended significantly more sessions and follow-ups than those in the SCBSCT group. Mediation analysis showed that the number of sessions attended by participants mediated in the relation between treatment condition and abstinence outcomes at the end of treatment and at 3-months follow-up, revealing that SCBSCT-BA was a predictor of session attendance and, through this relation, a predictor of abstinence outcomes. As no differences regarding treatment satisfaction were found between the two groups, this may be due to the fact that this treatment approach targets a broad range of smoking and mood-related aspects that provides participants with higher motivation to attend sessions. Additionally, the BA approach may have the capacity to retain participants in treatment, especially of those with higher risk of dropout (i.e., participants with depressive symptomatology or those with lower motivation to quit). Further research is needed to investigate these hypotheses, and also the predictors of treatment adherence and their relation to smoking and depression outcomes [69].

Our study has several limitations that should be mentioned. Firstly, missing data throughout the follow-up period could have affected the accuracy of results of the present study. Nevertheless, this is a frequent phenomenon in clinical trials addressing health-related outcomes such as smoking cessation [70]. In order to overcome this limitation, we used an analytic approach (Multiple Imputation) that has been recognized as a useful way to handle missing data and provide reliable results [53]. Using MI analyses, the AORs, although greater for the SCBSCT-BA condition, did not reach statistical significance at the 3-, 6-, and 12-month follow-ups. Participants attrition rates, and sample size may have influenced this result, so more research is needed to confirm long-term effects of BA. Secondly, the use of only two therapists delivering the treatment conditions could have introduced some biases. Nevertheless, both therapists had similar experience in smoking cessation treatment and were supervised during the study by two clinical psychologists. In previous studies where therapist effect was analyzed, no differences were found between them [2]. Thirdly, despite that both treatments were manualized and included the specific activities carried out session-by-session, the lack of treatment fidelity coding could be an important limitation. Treatment fidelity/integrity would allow a more clear interpretation of the obtained results [71], and consequently, future research should assess and verify the extent to which intervention content is delivered according to treatment manual specifications [72]. Fourthly, the results obtained may be not generalizable to smokers in the general population, as participants in this study enrolled voluntarily in a smoking cessation treatment, which implies a motivation to quit. Finally, the use of self-report questionnaires implies the typical limitations of all self-report assessment instruments (i.e., social desirability bias). However, we employed commonly used questionnaires that have shown their reliability and validity.

Despite these limitations, this work has strengths that deserve be highlighted. To date, this is the largest trial in terms of sample size to address the effects of BA integrated into a cognitive-behavioral smoking cessation treatment. In fact, abstinence rates at 6-, and 12-months follow-ups were of 30%, for the BA condition. Such rates are considerably high, as previous research showed that abstinence rates of smokers receiving pharmacotherapy at 6-, and 12-months follow-ups are between 14.5 and 28% [73]. In addition, we included two control conditions, one active group, which allows determine whether the BA approach was superior to a standard smoking cessation treatment; and one wait-list group, which allows to examine whether it was superior to the passage of time in which other variables, such as, for example, motivation or readiness to quit, could be acting. Another marked strength of this work is that we assessed study variables since the end of treatment to the 3-, 6-, and 12-month follow-ups in both active groups, which provided the opportunity to evaluate long-term treatment effects on abstinence rates and depressive symptoms. Moreover, SCBSCT-BA, which was delivered time-matched with the standard condition, increased abstinence rates of the standard condition. This suggests the potential cost-effectiveness of this approach. Finally, these results show that participants in general could benefit from a BA treatment approach that has been proven to be a strategy that promotes well-being among nonclinical populations [26].

In summary, the findings of the present study have several significant clinical implications derived from the relevance of depressive symptoms, regardless of their severity, and the reinforcement variables implicated in the smoking cessation process. Our results suggest that adding BA components to a cognitive behavioral treatment for quitting smoking implies greater abstinence rates in participants with a wide range of depressive symptoms, and also that smoking abstinence is related to a significant reduction of depressive symptoms. Therefore, the present findings support the benefit of BA as a component suitable for being integrated into smoking cessation treatments.

## Supporting information

S1 Text Consort checklist. (PDF)

S2 Text Bioethics committee study protocol Spanish. (PDF)

S3 Text Bioethics committee study protocol English translation. (PDF)

S4 Text Study protocol. (PDF)

S1 File Dataset. (SAV)
