# Supplementary material for: Cognitive-behavioral treatment with behavioral activation for smoking cessation: Randomized controlled trial
Source: PLoS One. 2019 Apr 8;14(4):e0214252. doi: 10.1371/journal.pone.0214252 (PMC6453447; doi:10.1371/journal.pone.0214252)
Supplement: S2 Text — (PDF) [file pone.0214252.s002.pdf]

**RESUMEN DEL PROYECTO DE INVESTIGACIÓN**

# EVALUACIÓN DE UN TRATAMIENTO PSICOLÓGICO PARA DEJAR DE FUMAR Y PARA LA MEJORA DEL ESTADO DE ÁNIMO DEPRESIVO

**INVESTIGADORES**

Elisardo Becoña Iglesias
Universidade de Santiago de Compostela. Facultade de Psicoloxía.
Departamento de Psicoloxía Clínica e Psicobioloxía

Carmen Senra Rivera
Universidade de Santiago de Compostela. Facultade de Psicoloxía.
Departamento de Psicoloxía Clínica e Psicobioloxía

Mª Carmen Míguez Varela
Universidade de Santiago de Compostela. Facultade de Psicoloxía.
Departamento de Psicoloxía Clínica e Psicobioloxía

Carmela Martínez Vispo
Universidade de Santiago de Compostela. Facultade de Psicoloxía.
Departamento de Psicoloxía Clínica e Psicobioloxía

Ana López Durán
Universidade de Santiago de Compostela. Facultade de Psicoloxía.
Departamento de Psicoloxía Clínica e Psicobioloxía

Elena Fernández del Río
Universidad de Zaragoza
Departamento de Psicología y Sociología

Rubén Rodríguez Cano
Universidade de Santiago de Compostela. Facultade de Psicoloxía.
Departamento de Psicoloxía Clínica e Psicobioloxía

**RESUMEN**

El consumo de tabaco sigue siendo en la actualidad la primera causa evitable de morbi-mortalidad. A pesar de que disponemos de tratamientos psicológicos eficaces para dejar de fumar, que obtienen buenos resultados, recientemente se ha observado una disminución de la eficacia al final del tratamiento, y unas mayores tasas de recaída, en las semanas o meses sucesivos.

Por otro lado, sabemos que existe una relación entre la depresión y el consumo de tabaco. De hecho, las personas con depresión tienen el doble de probabilidades de ser fumadoras. En los últimos diez años, en nuestro contexto hemos observado un incremento del número de fumadores con antecedentes de depresión (haber tenido en el pasado o que tienen depresión actualmente) que buscan tratamiento psicológico para dejar de fumar. Por este motivo nos planteamos que una intervención más intensiva, con componentes de activación conductual para el manejo del estado de ánimo depresivo, podría mejorar los resultados y, por tanto, la eficacia de la intervención psicológica para dejar de fumar.

El objetivo del presente estudio es evaluar la eficacia de un tratamiento psicológico para dejar de fumar con componentes específicos del procedimiento de activación conductual para el manejo del estado de ánimo depresivo.

Dispondremos de tres condiciones a las que 250 participantes fumadores serán asignados de manera aleatoria. La primera condición será la intervención estándar para dejar de fumar (Programa para dejar de fumar, n = 100), la segunda condición será la intervención para dejar de fumar con componentes para el manejo del estado de ánimo depresivo (Programa para dejar de fumar y mejora del estado de ánimo, n = 100) y la tercera condición será un grupo control de tratamiento demorado de tres meses (n = 50). Las dos primeras condiciones constarán de 8 sesiones grupales de intervención a razón de 1 sesión semanal de 1 hora de duración durante 8 semanas y seguimientos a los 3, 6 y 12 meses después de haber finalizado la intervención

Con este estudio se espera poder conocer si la incorporación de elementos específicos de manejo del estado de ánimo mejora los resultados de abstinencia de un tratamiento psicológico para dejar de fumar, así como evaluar si también se produce una mejora del estado de ánimo depresivo de los participantes.

## INTRODUCCIÓN

Actualmente, sabemos que fumar es la primera causa evitable de mortalidad y morbilidad (World Health Organization, 2009; U.S.D.H.H.S., 2014). En España, la prevalencia del consumo de tabaco es una de las más elevadas de Europa (OECD, 2014) y la mortalidad que se le atribuye se sitúa en más de 50.000 personas al año (Banegas et al., 2011; Hernández-García et al., 2010). En los últimos años, la prevalencia del consumo de tabaco ha experimentado un descenso importante, alcanzando la cifra más baja de los últimos 25 años, un 24% en 2013 (Instituto Nacional de Estadística, 2013). Sin embargo, todavía fuma un importante porcentaje de la población española y sigue siendo un factor de riesgo muy importante para la salud y la calidad de vida.

Fumar está asociado no solo con un peor estado de salud física, sino también psicológica y distintos estudios han encontrado una relación entre fumar y diferentes trastornos mentales (Becoña, 2004; Goodwin, Zvolensky, Keyes y Hasin, 2012). De hecho, las tasas de prevalencia de dependencia del tabaco son más elevadas en estos pacientes que en población general y, además, el grado de consumo y dependencia va asociado a la gravedad del trastorno mental (Royal College of Physicians, 2013).

Uno de los trastornos que con mayor frecuencia se ha asociado con fumar tabaco es la depresión, observándose una prevalencia muy elevada del consumo en las personas que presentan este trastorno (Lasser, Boyd, Woolhandler, Himmelstein, McCormick y Bor, 2000; Tjora, Hetland, Aarø, Wold, Wiium y Øverland, 2014). Además, suelen mostrar mayor dependencia de la nicotina, más cambios negativos en el estado de ánimo debidos al síndrome de abstinencia, mayores porcentajes de recaída después de dejar de fumar y mayor riesgo de morbi-mortalidad atribuible al consumo de tabaco (Weinberger, Mazure, Morlett y McKee, 2013; Zvolensky, Bakhshaie, Sheffer, Perez y Goodwin, 2015), llegando a indicarse que el tabaco es el responsable del 50% de las muertes en pacientes con trastornos depresivos (Callaghan et al., 2014).

En la actualidad, aunque disponemos de intervenciones psicológicas eficaces para dejar de fumar, se ha señalado que las tasas de abstinencia en fumadores con depresión actual son significativamente más bajas que las de los fumadores sin antecedentes de trastorno depresivo mayor, debido quizás a que experimentan un síndrome de abstinencia más intenso o a que su estado de ánimo empeora cuando dejan de fumar (Gierisch, Bastian, Calhoun, McDuffie y Williams, 2012). Algunas investigaciones han sugerido que al dejar de fumar también podría incrementarse la probabilidad de recurrencia de un trastorno depresivo mayor en personas con historia

pasada de depresión (Becoña, 2004; Hughes, 2007). Por tanto, tal y como apuntan algunos investigadores, este tipo de población podría requerir una intervención más intensiva, ya que suelen presentar una mayor dependencia de la nicotina, es más probable que sufran cambios negativos en el estado de ánimo debidos al síndrome de abstinencia, tienen mayores probabilidades de recaer y también un mayor riesgo de morbimortalidad relacionada con el tabaco (Weinberger et al., 2013).

Por otro lado, la técnica psicológica de activación conductual podría definirse como una intervención estructurada y breve, aunque flexible, enfocada a lograr que las personas con sintomatología depresiva realicen cambios en sus conductas y modifiquen su entorno para que se restablezca su contacto con fuentes de reforzamiento positivo (Cuipjers, Van Straten y Warmerdam, 2007). La activación conductual se fundamenta en la idea de que las acciones y reacciones que caracterizan a las personas deprimidas juegan un papel significativo en el estado de ánimo. Esta intervención ha demostrado su eficacia en el tratamiento de la depresión en diversos estudios y se considera un tratamiento empíricamente validado para la depresión, resultando una modalidad de tratamiento efectiva en diferentes muestras clínicas y diversos contextos (Cuijpers et al., 2007; Ekers, Webster, Van Straten, Cuijpers, Richards y Gilbody, 2014; Hopko, Bell, Armento, Hunt y Lejuez, 2005; MacPherson et al, 2010). Además, es una de las intervenciones recomendadas por la Organización Mundial de la Salud (2013) para tratar la depresión. Por tanto, la activación conductual es un tratamiento eficaz para la depresión y resulta un enfoque relativamente sencillo y eficiente en el tiempo que puede ser adecuado como intervención psicológica para una amplia variedad de pacientes.

En los últimos diez años, y específicamente en nuestro contexto, se ha observado un incremento del número de fumadores con antecedentes de depresión (haber tenido en el pasado o tener actualmente un episodio de depresión mayor) que buscan tratamiento para dejar de fumar (Becoña, López-Durán, del Río y Martínez, 2014). Por este motivo nos planteamos que una intervención más intensiva, con componentes de activación conductual para el manejo del estado de ánimo depresivo, podría mejorar los resultados de abstinencia y, por tanto, la eficacia de la intervención psicológica para dejar de fumar.

## OBJETIVOS

Teniendo en cuenta que fumar cigarrillos es la primera causa evitable de muerte en España, al igual que en el resto de los países desarrollados, y dado que dejar de

fumar está relacionado con tener sintomatología depresiva y con padecer depresión, es de gran relevancia conocer mejor esta relación, así como incorporar nuevos elementos a los tratamientos psicológicos para dejar de fumar, ya que esto permitiría mejorar las tasas de abstinencia y prevenir la recaída.

Por ello, el objetivo del presente estudio es la elaboración y evaluación de una intervención psicológica para el manejo de los estados emocionales depresivos basado en la técnica de activación conductual en el marco de un tratamiento psicológico para dejar de fumar. También es objeto de interés conocer si la incorporación de dichos elementos está asociada con mejores resultados en las tasas de abstinencia a corto y a largo plazo, así como con una mejoría en el estado de ánimo de los participantes.

**Objetivos generales:**

1. Diseñar y elaborar una intervención psicológica eficaz para dejar de fumar que incluya elementos para el manejo de la sintomatología depresiva a través de la técnica de la activación conductual.
2. Analizar la eficacia de dicha intervención a corto plazo (final tratamiento) y a largo plazo (seguimiento de 3, 6 y 12 meses), mediante un estudio de asignación aleatoria a tres grupos: a) tratamiento estándar (programa para dejar de fumar), b) tratamiento estándar más manejo del estado de ánimo, y c) grupo control (de tratamiento demorado).
3. Evaluar si la intervención psicológica para dejar de fumar y para la mejora del estado de ánimo depresivo, mejora la sintomatología depresiva a corto (final del tratamiento) y a largo plazo (seguimiento de 3, 6 y 12 meses).

## METODOLOGÍA

### Muestra

La muestra estará compuesta por fumadores que acuden a la Unidad de Tabaquismo y Trastornos Adictivos de la USC que demandan tratamiento para dejar de fumar. Todos ellos pueden demandar información acerca del programa para dejar de fumar después de diversas comunicaciones a la prensa, carteles situados en distintos puntos de la ciudad y de los centros de salud, a través de otros fumadores que hayan acudido previamente al tratamiento o debido a la remisión por parte de su médico de atención primaria u otros servicios especializados del sistema sanitario.

Los 250 participantes que cumplan los criterios de inclusión y exclusión serán asignados al azar a uno de los tres grupos siguientes:

1. Tratamiento psicológico para dejar de fumar estándar (n = 100).
2. Tratamiento psicológico para dejar de fumar más componentes para la mejora del estado de ánimo depresivo (n = 100).
3. Grupo de control de tratamiento demorado de 3 meses (n = 50).

Más adelante se describen los mismos.

***Criterios de inclusión/exclusión***

***Inclusión***

- Tener 18 o más años.
- Deseo de participar voluntariamente en el tratamiento psicológico *Programa para dejar de fumar.*
- Presentar un consumo mínimo de 8 cigarrillos diarios antes del inicio del tratamiento.
- Cumplimentar el consentimiento informado

***Exclusión***

- Tener un diagnóstico de trastorno mental grave (trastorno bipolar y/o trastorno psicótico).
- Dependencia concurrente de otras sustancias (cannabis, cocaína, heroína, etc.).
- Haber participado en el tratamiento psicológico *Programa para dejar de fumar* u otro tratamiento psicológico eficaz para dejar de fumar durante el año previo.
- Haber recibido otro tipo de tratamiento efectivo para dejar de fumar en el último año (chicles o parches de nicotina, bupropión, vareniclina).
- Padecer alguna patología que implique un alto riesgo vital para la persona y que precise una intervención inmediata en formato individual (ej. infarto de miocardio reciente, neumotórax, etc.).
- Fumar productos derivados del tabaco diferentes a los cigarrillos (ej. puros o puritos).

**Evaluación**

Antes del inicio del tratamiento para dejar de fumar todos los fumadores participantes en el estudio realizarán dos sesiones presenciales de evaluación pretratamiento en la que se les administrarán los siguientes cuestionarios.

- *Cuestionario sobre el hábito de fumar* (Becoña, 1994): cuestionario formado por 59 preguntas que analizan distintos aspectos de la conducta de fumar: variables demográficas (edad, sexo, estado civil, estudios, profesión, clase social e ingresos económicos), consumo de tabaco (consumo actual y pasado de cigarrillos y de otros productos del tabaco), intentos previos de abandono o reducción de cigarrillos, razones para dejar de fumar en intentos previos, y creencias sobre la incidencia del tabaco en la salud.
- Estadios de cambio: se utilizan dos ítems para evaluar el estadio de cambio en el que se encuentra el fumador en el momento de la demanda de tratamiento según el modelo transteórico de Prochaska y DiClemente (1983). Este modelo entiende las conductas adictivas como un continuo en el que la persona pasa por distintas fases de recuperación y recaída hasta que finalmente consigue la abstinencia a corto, medio y largo plazo. Los estadios que considera este modelo son los de: *precontemplación* (el fumador empieza a contemplar su conducta como un problema y no tiene intención de dejar de fumar al menos en los próximos 6 meses), *contemplación* (el fumador empieza a contemplar su conducta como un problema, busca activamente información y se plantea seriamente la posibilidad de dejar el tabaco en los próximos 6 meses), *preparación para la acción* (el fumador ya ha tomado la decisión de modificar su conducta en los siguientes 30 días, además para considerar que se encuentra en este estadio tiene que haber hecho al menos un intento de abandono de 24 horas de duración en el último año), *acción* (el fumador deja de fumar y se mantiene sin consumir cigarrillos como mínimo 24 horas), *mantenimiento* (lleva 6 meses o más sin fumar) y *finalización* (el exfumador lleva 5 años o más sin fumar, observándose una ausencia del deseo de fumar cigarrillos en cualquier situación que se le presente y la confianza de no fumar en todas las situaciones problemáticas es del cien por cien).
- *Test de Fargerström de Depedencia de la Nicotina (Fagerström Test for Nicotine Dependence,* FTND; Heatherton, Kozlowski, Frecker y Fagerström, 1991; adaptación al castellano de Becoña, 1994 y Becoña y Vázquez, 1998): formado por 6 ítems de dos a cuatro alternativas de respuesta con un punto de

corte para la dependencia de la nicotina de 6 (Fagerström et al., 1996). Este test ha sido validado con medidas fisiológicas del contenido de nicotina en sangre, por lo que permite que sea utilizado para medir el grado de dependencia fisiológica y se ha demostrado que la relación entre la puntuación en este cuestionario y las medidas fisiológicas de fumar son altas (Becoña y García, 1995). Su utilización en investigación es habitual ya que es uno de los cuestionarios de referencia para la evaluación de los fumadores.

- *Escala del Síndrome de Dependencia de la Nicotina (Nicotine Dependence Syndrome Scale*, NDSS; Shiffman, Waters y Hickcox, 2004; adaptación española de Becoña, López, Fernández del Río, Míguez y Castro, 2010): cuestionario multidimensional basado en la conceptualización de Edwards de la dependencia de sustancias como un síndrome (Edwards, 1986; Edwards y Gross, 1976). Se trata de una escala autoinformada de 19 ítems que evalúan la dependencia de la nicotina.
- *Escala de Autoeficacia/Tentación de Fumar* (Velicer et al., 1990). Consta de 9 items que evalúan la tentación de fumar en diferentes situaciones: situación de afecto positivo/situación social, situaciones de afecto positivo y situaciones de hábito/craving.
- *Minnesota Nicotine Withdrawal Scale* (MNWS; Hughes y Hatsukami, 1986). Consta de 8 ítems que evalúan los síntomas del síndrome de abstinencia: depresión, insomnio, irritabilidad/frustración/ira, ansiedad, dificultad de concentración, intranquilidad, incremento del apetito/ganancia de peso y craving (deseo o necesidad de fumar; el cual se considera de forma independiente).
- *Entrevista Clínica Estructurada de dependencia del tabaco según el DSM-5*. Aplicar los criterios diagnósticos del trastorno por consumo de tabaco.
- *Cuestionario de Screening de Episodios de Depresión Mayor (*MDE, Muñoz, 1998). Instrumento de detección de episodio depresivo mayor en el pasado y/o en el momento actual. Se basa en los criterios diagnósticos recogidos en el DSM para el diagnóstico de episodios de depresión mayor.
- *Escala de Depresión de Hamilton* (HRDS; Hamilton, 1960). Instrumento diseñado para evaluar cuantitativamente la gravedad de los síntomas depresivos y monitorizar los cambios.

- *Inventario de Depresión de Beck II (BDI-II)* (Beck, Steer y Brown, 1996; Sanz, Perdigón y Vásquez, 2003). Se trata de una escala de tipo likert que consta de 21 ítems de autoinforme, construida con el propósito de medir los niveles de depresión en población normal o psiquiátrica, a partir de los 13 años. Este inventario persigue describir la presencia y el grado de síntomas depresivos en dichas poblaciones, sin pretender establecer un diagnóstico clínico (Beck et al., 1996). La puntuación total máxima que se puede obtener es de 63.
- EROS *(Environmental Reward Observation Scale,* Armento y Hopko, 2007; Barraca y Pérez-Álvarez, 2010). Es un instrumento breve de 10 ítems desarrollado para obtener una auto-evaluación objetiva del grado en que el entorno resulta reforzante para la persona. Una puntuación alta se vincula con un incremento de las conductas y del afecto positivo como consecuencia de experiencias reforzantes desde el medio.
- BADS (*Behavioral Activation for Depression Scale*, Kanter, Mulick, Busch, Berlin y Martell, 2007; Barraca, Pérez-Álvarez y Bleda, 2011). Es un cuestionario de 25 ítems elaborado para medir cuatro dimensiones básicas para el modelo de la activación conductual: Activación, Evitación/Rumia, Afectación del trabajo/actividad y Afectación de la vida social.
- Escala de soledad de UCLA (Version 3) (Russell, 1996): La tercera versión de la escala ha sido adaptada a nuestro país por Expósito y Moya (1993). Dicha escala consta de 20 afirmaciones que reflejan el grado de soledad del individuo. Esta escala ha sido la más ampliamente utilizada en los estudios sobre soledad y posee buenos índices de fiabilidad (Shaver y Brennan, 1991). Las puntuaciones obtenidas en la Escala de Soledad de UCLA se han relacionado con la conducta social, los patrones de atribución y los sentimientos de abandono, depresión, vacío, indefensión, aislamiento, entre otros. Conforme mayor es la puntuación obtenida en la escala, mayor es el nivel de soledad, pudiendo oscilar las puntuaciones entre 20 y 80.
- *Escala de Respuestas Rumiativas* (RRS, Nolen-Hoeksema, Larson y Grayson, 1999; Hervás; 2008). Consta de 22 ítems autoinformados. En 2003, Treynor et al. depuraron la *RRS* eliminando los 12 ítems que se solapaban con síntomas depresivos lo que dio lugar a una nueva versión de 10 ítems. Un análisis

factorial exploratorio con estos 10 ítems confirmó una solución factorial de dos factores: rumiación *brooding* (5 ítems) y rumiación *reflection* (5 ítems).

- *Autorregistros.* Se trata de unas hojas que se les proporcionan a los participantes con el fin de que conozcan en profundidad su conducta de fumar. En esta hoja, que debe llevar junto con el paquete de tabaco en todo momento y que debe cubrir diariamente, se incluyen los siguientes apartados: número de cigarrillos (hasta 40 cigarrillos por hoja), hora en la que fuma el cigarrillo, placer que le provoca (de 0 a 10, siendo 0 el mínimo placer y 10 el máximo) y situación en la que se encuentra.
- *Evaluación del monóxido de carbono (CO) en aire espirado.* La evaluación del CO a través del aire espirado es una medida fácil de usar, no invasiva y que proporciona un feedback inmediato, además de no requerir de personal especializado para su aplicación. Se utilizará el Micro+ Smokerslyzer ® (Bedfont Scientific Instruments Ltd, Sittingbourne, Kent, Reino Unido). Este aparato proporciona una lectura digital de las partículas por millón (ppm) de CO en el aire espirado y puede transformarse en el porcentaje equivalente de COHb (carboxihemoglobina) en sangre.
- *Cuestionario de final del tratamiento.* En la última sesión de tratamiento los participantes cumplimentan el *Cuestionario de evaluación de final de tratamiento* (Becoña y Míguez, 1995) en el que se evalúan los siguientes aspectos: consumo actual de cigarrillos (número de cigarrillos fumados ese día y el anterior y marca consumida); fecha para dejar de fumar (en caso de no haber dejado de fumar se les pregunta qué día tienen previsto realizar el abandono definitivo); apoyo recibido por parte de las personas de su entorno; mejorías físicas y psicológicas experimentadas desde el inicio del *Programa para dejar de fumar*; posible empeoramiento durante las semanas de aplicación del tratamiento; escala de síntomas de abstinencia del tabaco de Hughes y Hatsukami (1986); valoración de la confianza en mantenerse sin fumar en los próximos 6 meses en una escala de 0 (nada) a 10 (máximo); valoración de la satisfacción con los servicios recibidos (excelente, buena, regular o mala) mediante el Cuestionario de satisfacción del cliente (CSQ-8) de Larsen, Attkinson, Hargreaves y Nguyen (1979); comentarios que la persona deseen hacer sobre el programa que ha seguido y su proceso de abandono.

- *Cuestionarios de seguimiento*. Autoinforme donde se recogen los datos acerca de la abstinencia y/o recaída a los 3, 6 y 12 meses de seguimiento.

**Procedimiento**

Dispondremos de tres condiciones a las que 250 participantes serán asignados de manera aleatoria (secuencia 2.2.1.). La primera condición será la intervención estándar para dejar de fumar (Programa para dejar de fumar, n = 100), la segunda condición será la intervención para dejar de fumar con componentes para el manejo del estado de ánimo (Programa para dejar de fumar y mejora del estado de ánimo, n = 100) y la tercera condición será un grupo control demorado de lista de espera de tres meses (n = 50).

Las dos primeras condiciones constarán de 8 sesiones grupales de intervención a razón de 1 sesión semanal de 1 hora de duración durante 8 semanas y seguimientos a los 3, 6 y 12 meses de haber finalizado el programa.

Los participantes que forman parte del grupo control de tratamiento demorado de tres meses serán evaluados al inicio y a los tres meses de espera, de forma que así podemos comparar los tres grupos al final del tratamiento.

**Descripción de los programas de tratamiento psicológico para dejar de fumar**

A continuación se describen los componentes y procedimientos empleados en cada sesión de manera específica. Una descripción detallada de los componentes de las dos condiciones de tratamiento sesión a sesión puede encontrarse en las tablas 1 y 2.

***Programa Para Dejar De Fumar Estándar***

El tratamiento estándar para dejar de fumar cognitivo-conductual es un tratamiento manualizado, denominado "Programa para dejar de fumar". Los componentes del tratamiento son: contrato terapéutico, autorregistros y representación gráfica del consumo de cigarrillos, información sobre el tabaco, reducción de la nicotina (cambio de marcas de cigarrillos cada semana, disminución progresiva de la ingesta de nicotina y alquitrán), control de estímulos, actividades para prevenir el síndrome de abstinencia, feedback fisiológico (CO en el aire expirado) sobre el consumo de cigarrillos, y estrategias de prevención de recaída (entrenamiento en aserción, entrenamiento de resolución de problemas, cambios en pensamientos erróneos relacionados con el tabaco, manejo de la ansiedad y la ira, ejercicio, control de peso y

auto-refuerzo). El tratamiento se administrará en ocho sesiones de 60 minutos durante 8 semanas consecutivas.

Tabla 1
Resumen sesión por sesión de los componentes del tratamiento cognitivo-conductual para dejar de fumar estándar (CBSCT)

| Sesión | Componentes |
|---|---|
| 1 | Resumen de las características del tratamiento<br>Justificación del tratamiento para dejar de fumar<br>Revisar los autorregistros (apuntar los cigarrillos fumados y los antecedentes y consecuentes de la conducta de fumar)<br>Indicaciones sobre cómo representar gráficamente el número de cigarrillos / día<br>Razones para fumar y para dejar de fumar<br>Historia previa y experiencias relacionadas con los intentos para dejar de fumar<br>Explicar y proporcionar materiales escritos sobre el tabaco, la dependencia a la nicotina, las consecuencias para la salud del consumo de tabaco y los beneficios de dejar de fumar.<br>Explicar la reducción de la ingesta de nicotina a través del cambio de marca.<br>Feedback fisiológico (CO)<br>Como tarea:<br>- Cambio de marca<br>- Comunicarle al menos una persona (familia, amigo, compañero de trabajo, etc.) que va a intentar dejar de fumar en los próximos 30 días.<br>- No fumar más cigarrillos del promedio de los fumados la semana anterior.<br>- Dejar un tercio del cigarrillo sin fumar.<br>- Rechazar ofrecimientos de cigarrillos. |
| 2 | Comprobar el cumplimiento de las tarea y la reducción de la ingesta de nicotina<br>Continuar realizando los autorregistros y analizar la conducta de fumar durante la semana. |

| | |
|---|---|
| | Feedback fisiológico (CO)<br>Discutir las dificultades surgidas con el cambio de marca.<br>Nuevo cambio de marca y reducción del número de cigarrillos.<br>Revisar la importancia del apoyo social.<br>Introducir la técnica de control de estímulos para eliminar situaciones asociadas a la conducta de fumar.<br>El síndrome de abstinencia de la nicotina y estrategias para evitarlo.<br>Ejercicios de respiración y técnicas de relajación (práctica como tarea). |
| 3 | Comprobación de la reducción de la ingesta de nicotina, la reducción del consumo de cigarrillos y el cumplimiento del control de estímulos<br>Comprobar el cumplimiento de los ejercicios de respiración y estrategias para evitar los síntomas del síndrome de abstinencia.<br>Feedback fisiológico (CO)<br>Continuar realizando los autorregistros y analizar la conducta de fumar.<br>Nuevo cambio de marca y reducción del número de cigarrillos.<br>Continuar con la técnica de control de estímulos.<br>Explicar y proporcionar materiales escritos para el control de peso y para promover el ejercicio físico.<br>Continuar con los ejercicios de respiración y las estrategias para evitar los síntomas de abstinencia. |
| 4 | Comprobación de la reducción de la ingesta de nicotina, la reducción del consumo de cigarrillos y el cumplimiento del control de estímulos<br>Comprobar el cumplimiento de los ejercicios de respiración y estrategias para evitar los síntomas del síndrome de abstinencia.<br>Feedback fisiológico (CO)<br>Continuar realizando los autorregistros y analizar la conducta de fumar.<br>Nuevo cambio de marca y reducción del número de cigarrillos.<br>Continuar con la técnica de control de estímulos.<br>Estrategias de manejo del estrés y la ansiedad.<br>Entrenamiento en resolución de problemas |
| 5 | Comprobación de la reducción de la ingesta de nicotina, la reducción del consumo de cigarrillos y el cumplimiento del control de estímulos<br>Feedback fisiológico (CO) |

| | |
|---|---|
| | Continuar realizando los autorregistros y analizar la conducta de fumar.<br>Nuevo cambio de marca y reducción del número de cigarrillos.<br>Manejo de la ansiedad y la ira.<br>Autorefuerzo<br>Discusión acerca de conceptos erróneos relacionados con el tabaco. |
| 6 | La experiencia de dejar de fumar y los síntomas del síndrome de abstinencia.<br>Feedback fisiológico (CO)<br>Discutir y planificar estrategias para situaciones de lato riesgo de caíada o recaída Factores motivadores para mantener la abstinencia.<br>Beneficios de dejar de fumar<br>Barreras comunes para mantener la abstinencia. |
| 7 | La experiencia de dejar de fumar y los síntomas del síndrome de abstinencia.<br>Feedback fisiológico (CO)<br>Discutir y planificar estrategias para situaciones de lato riesgo de caíada o recaída Factores motivadores para mantener la abstinencia.<br>Beneficios de dejar de fumar<br>Estrategias de prevención de la recaída. |
| 8 | Feedback fisiológico (CO)<br>Gestionando el futuro como ex fumadores.<br>Fomento del mantenimiento de la abstinencia.<br>Estrategias a usar en caso de caídas o recaídas.<br>Revisar los factores motivadores, los cambios en el estilo de vida, la mejora de la salud física y psicológica al dejar de fumar.<br>Conclusión y manejo de posibles obstáculos para mantenerse sin fumar |

***Programa para dejar de fumar estándar y la activación conductual***

La activación conductual se aplicará junto con el tratamiento cognitivo-conductual estándar para dejar de fumar descrito anteriormente. Los componentes del tratamiento son los mencionados anteriormente junto con los siguientes: análisis de la relación entre conducta y estado de ánimo, identificación de situaciones y comportamientos que disminuyen el estado de ánimo, identificación de conductas de evitación, identificación de los pensamientos de rumiación y preocupación, registro de actividades diarias agradables, programación de actividades para aumentar la participación en actividades gratificantes y para reducir los patrones conductuales de

evitación. El tratamiento se administrará en ocho sesiones de 60 minutos durante 8 semanas consecutivas.

Tabla 2

Resumen sesión por sesión de los componentes del tratamiento cognitivo-conductual para dejar de fumar estándard y activación conductual (CBSCT + AC)

| Sesión | Componentes |
|---|---|
| 1 | Resumen de las características del tratamiento<br>Justificación del tratamiento para dejar de fumar<br>Revisar los autorregistros (apuntar los cigarrillos fumados y los antecedentes y consecuentes de la conducta de fumar)<br>Indicaciones sobre cómo representar gráficamente el número de cigarrillos / día<br>Razones para fumar y para dejar de fumar<br>Historia previa y experiencias relacionadas con los intentos para dejar de fumar<br>Explicar y proporcionar materiales escritos sobre el tabaco, la dependencia a la nicotina, las consecuencias para la salud del consumo de tabaco y los beneficios de dejar de fumar.<br>Explicar la reducción de la ingesta de nicotina a través del cambio de marca.<br>Feedback fisiológico (CO)<br>Como tarea:<br>- Cambio de marca<br>- Comunicarle al menos una persona (familia, amigo, compañero de trabajo, etc.) que va a intentar dejar de fumar en los próximos 30 días.<br>- No fumar más cigarrillos del promedio de los fumados la semana anterior.<br>- Dejar un tercio del cigarrillo sin fumar.<br>- Rechazar ofrecimientos de cigarrillos. |
| 2 | Comprobar el cumplimiento de las tarea y la reducción de la ingesta de nicotina<br>Continuar realizando los autorregistros y analizar la conducta de fumar durante la semana. |

| | |
|---|---|
| | Feedback fisiológico (CO)<br>Discutir las dificultades surgidas con el cambio de marca.<br>Nuevo cambio de marca y reducción del número de cigarrillos.<br>Revisar la importancia del apoyo social.<br>Introducir la técnica de control de estímulos para eliminar situaciones asociadas a la conducta de fumar.<br>El síndrome de abstinencia de la nicotina y estrategias para evitarlo.<br>Ejercicios de respiración y técnicas de relajación (práctica como tarea). |
| 3 | Comprobación de la reducción de la ingesta de nicotina, la reducción del consumo de cigarrillos y el cumplimiento del control de estímulos<br>Comprobar el cumplimiento de los ejercicios de respiración y estrategias para evitar los síntomas del síndrome de abstinencia.<br>Feedback fisiológico (CO)<br>Continuar realizando los autorregistros y analizar la conducta de fumar.<br>Nuevo cambio de marca y reducción del número de cigarrillos.<br>Continuar con la técnica de control de estímulos.<br>Explicar y proporcionar materiales escritos para el control de peso y para promover el ejercicio físico.<br>Continuar con los ejercicios de respiración y las estrategias para evitar los síntomas de abstinencia.<br>Justificación de la influencia del estado de ánimo en el abandono del hábito de fumar (proporcionar materiales escritos)<br>Para casa: autorregistro de actividades diarias. |
| 4 | Comprobación de la reducción de la ingesta de nicotina, la reducción del consumo de cigarrillos y el cumplimiento del control de estímulos<br>Comprobar el cumplimiento de los ejercicios de respiración y estrategias para evitar los síntomas del síndrome de abstinencia.<br>Feedback fisiológico (CO)<br>Continuar realizando los autorregistros y analizar la conducta de fumar.<br>Nuevo cambio de marca y reducción del número de cigarrillos.<br>Continuar con la técnica de control de estímulos.<br>Estrategias de manejo del estrés y la ansiedad.<br>Revisión del autorregistro de actividades y reconocimiento de patrones |

| | |
|---|---|
| | conductuales relacionados con el estado de ánimo y la forma en que participar en actividades gratificantes e importantes puede afectar su estado de ánimo general<br>Tarea: continuar con la programación de actividades, crear una lista de actividades placenteras y elegir una para hacer durante la semana |
| 5 | Comprobación de la reducción de la ingesta de nicotina, la reducción del consumo de cigarrillos y el cumplimiento del control de estímulos<br>Feedback fisiológico (CO)<br>Continuar realizando los autorregistros y analizar la conducta de fumar.<br>Nuevo cambio de marca y reducción del número de cigarrillos.<br>Manejo de la ansiedad y la ira.<br>Autorefuerzo<br>Discusión acerca de conceptos erróneos relacionados con el tabaco.<br>Entrenamiento para resolver problemas<br>Reconocer el comportamiento de evitación y el impacto en el estado de ánimo.<br>Programación de actividades y participación en 2 actividades agradables / semana. |
| 6 | La experiencia de dejar de fumar y los síntomas del síndrome de abstinencia.<br>Feedback fisiológico (CO)<br>Discutir y planificar estrategias para situaciones de lato riesgo de caíada o recaída Factores motivadores para mantener la abstinencia.<br>Beneficios de dejar de fumar<br>Barreras comunes para mantener la abstinencia.<br>Pensamientos de rumiación y el proceso para dejar de fumar y la recaída.<br>Comprobación de que se han realizado las tareas de programación de actividades<br>Programación de actividades para la próxima semana y participación en 2 actividades agradables por semana. |
| 7 | La experiencia de dejar de fumar y los síntomas del síndrome de abstinencia.<br>Feedback fisiológico (CO)<br>Discutir y planificar estrategias para situaciones de lato riesgo de caída o recaída |

| | |
|---|---|
| | Factores motivadores para mantener la abstinencia.<br>Beneficios de dejar de fumar<br>Revisión de las estrategias de activación conductual y su influencia en el estado de ánimo<br>Revisión de las conductas de evitación y los pensamientos de rumiación.<br>Estrategias de prevención de la recaída. |
| 8 | Feedback fisiológico (CO)<br>Gestionando el futuro como ex fumadores.<br>Fomento del mantenimiento de la abstinencia.<br>Estrategias a usar en caso de caídas o recaídas.<br>Revisar los factores motivadores, los cambios en el estilo de vida, la mejora de la salud física y psicológica al dejar de fumar.<br>Revisar estrategias de activación conductual<br>Conclusión y manejo de posibles obstáculos para mantenerse sin fumar |

***Grupo control de lista de espera***

Será un grupo de control de tratamiento demorado por un período de 3 meses. Después del período de 3 meses, se realizará otra evaluación y luego se ofrecerá a los participantes el acceso a un tratamiento para dejar de fumar.

**Procedimiento de realización de las intervenciones psicológicas**

- Se realizarán dos sesiones de evaluación antes del comienzo del tratamiento.
- En ambos grupos se aplicarán 8 sesiones, a razón de 1 sesión semanal de 60 minutos, a lo largo de 8 semanas.
- La aplicación de la intervención psicológica será realizada por un psicólogo clínico o psicólogo general sanitario. Estos profesionales recibirán entrenamiento en cada uno de los tratamientos a aplicar y serán ciegos al otro tratamiento.
- Se volverá a realizar una evaluación al final del tratamiento de las variables relevantes para el estudio.
- Se realizarán seguimientos, y sus correspondientes evaluaciones, a los 3, 6 y 12 meses después de haber finalizado la intervención.
- La evaluación de la abstinencia se hará en todos los casos con la información proporcionada por la persona, validada con una medida objetiva: monóxido de carbono en el aire espirado, evaluada a través de un Micro+ Smokerlizer. Se considera abstinente

al final del tratamiento no haber fumado durante ≥ 24 horas al final del tratamiento y un CO de ≤ 10 partículas por millón (ppm). Respecto a los seguimientos, se utilizará tanto el criterio de abstinencia puntual (Velicer et al., 1992) como el de abstinencia continua (West et al., 2005). Un participante será considerado abstinente a los 3 meses de seguimiento si informa de no haber fumado, ni siquiera una calada ≥ 7 días antes del día de seguimiento y tiene un CO de ≤ 10 ppm. En los seguimientos de 6 y 12 meses, los participantes se considerarán abstinentes si informan no haber fumado, ni siquiera una calada durante ≥ 30 días antes del día de seguimiento, y tienen un CO ≤ 10 ppm. En aquellos casos en los que no sea posible localizar a los participantes, se los considerará fumadores en el seguimiento correspondiente.

## ANÁLISIS DE DATOS

Para el análisis estadístico de los datos se utilizará el paquete estadístico IBM SPSS Statistics. Todos los análisis se realizarán utilizando el principio intención a tratar. Realizaremos un análisis descriptivo para resumir las características de la muestra total y las características de los participantes en cada uno de los tres grupos. El análisis principal será una comparación entre los grupos activos y el grupo de control de los porcentajes de abstinencia al final del tratamiento, y entre el grupo de intervención de activación conductual y el grupo de intervención estándar al final del tratamiento, y en los seguimientos a los 3, 6, y 12 meses, a través de pruebas de chi-cuadrado y odds ratios con intervalos de confianza del 95%. También se realizarán pruebas t, análisis de regresión, ANOVA de medidas repetidas y análisis de mediación.

## RESULTADOS QUE SE ESPERAN OBTENER CON ESTE ESTUDIO

- Diseñar y elaborar un tratamiento psicológico eficaz para dejar de fumar que incluya elementos para el manejo de la sintomatología depresiva a través de la técnica de la activación conductual.
- Analizar la eficacia de dicho tratamiento a corto plazo (final tratamiento) y a largo plazo (seguimiento de 3, 6 y 12 meses), mediante un estudio de asignación aleatoria a tres grupos: a) tratamiento estándar (programa para dejar de fumar), b) tratamiento estándar más manejo del estado de ánimo, y c) grupo control (de tratamiento demorado).
- Evaluar si el tratamiento psicológico para dejar de fumar y para la mejora del estado de ánimo depresivo, mejora la sintomatología depresiva a corto (final del

tratamiento) y a largo plazo (seguimiento de 3, 6 y 12 meses) y otras variables relacionadas con el consumo de tabaco como craving, autoeficacia y síndrome de abstinencia.

**Resultados esperados específicos:**

1. Los dos tratamientos activos obtendrán mejores resultados con respecto a la abstinencia que la condición de control, al final del tratamiento.
2. El tratamiento de manejo del estado ánimo depresivo será más eficaz en cuanto al porcentaje de abstinencia que el tratamiento estándar, al final del tratamiento y en los seguimientos de 3, 6 y 12 meses.
3. Los dos tratamientos activos lograrán reducciones significativas en las escalas que evalúan sintomatología depresiva (Inventario de Depresión de Beck II, Escala de Depresión de Hamilton) en relación a la condición de control, al final del tratamiento.
4. Los participantes del grupo de manejo del estado de ánimo depresivo lograrán reducciones significativas en las escalas que evalúan sintomatología depresiva (Inventario de Depresión de Beck II, Escala de Depresión de Hamilton) en relación a los participantes en el tratamiento estándar, al final del tratamiento y en los seguimientos de 3, 6 y 12 meses.
5. Los dos tratamientos activos lograrán una mejora significativa en relación al grupo control en las variables de craving, autoeficacia y síndrome de abstinencia, al final del tratamiento.
6. Los participantes del grupo de manejo del estado de ánimo depresivo tendrán una mejora significativa en relación al grupo estándar en las variables de craving, autoeficacia y síndrome de abstinencia, al final del tratamiento y en los seguimientos de 3, 6 y 12 meses.
7. Los dos tratamientos activos lograrán una mejora significativa en relación al grupo control en las escalas que evalúan activación conductual (Escala Observacional de Refuerzo Ambiental**,** *EROS*; Escala de Activación Conductual para la Depresión, *BADS*), al final del tratamiento.
8. Los participantes en el grupo de manejo del estado de ánimo depresivo lograrán una mejora significativa en relación al grupo de tratamiento estándar en las escalas que evalúan activación conductual (*EROS*; *BADS*), al final del tratamiento y en los seguimientos de 3, 6 y 12 meses.

**REFERENCIAS**

Armento, M. E.y Hopko, D. R. (2007). The environmental reward observation scale (EROS): development, validity, and reliability. *Behavior Therapy, 38*, 107-119.

Barraca, J., Pérez-Álvarez, M., & Bleda, J. H. L. (2011). Avoidance and activation as keys to depression: Adaptation of the Behavioral Activation for Depression Scale in a Spanish sample. *Spanish Journal of Psychology*, *14*, 998-1009.

Barraca, J. y Pérez-Álvarez, M. (2010). Spanish adaptation of the Environmental Reward Observation Scale (EROS), *Anxiety and Stress*, *16*, 95-107.

Banegas, J. R., Díez-Gañán, L., Bañuelos-Marco, B., González-Enríquez, J., Villar-Álvarez, F., Martín-Moreno, J. M., … Jiménez-Ruíz, C. (2011). Mortalidad atribuíble al consumo de tabaco en España en 2006. *Medicina Clínica, 136,* 97-102.

Banegas, J. R., Díez, L., González, J., Villar, F. y Rodríguez-Artalejo, F. (2005). La mortalidad atribuible al tabaquismo comienza a descender en España. *Medicina Clínica, 124,* 769-771.

Beck, A. T., Steer, R. y Brown, G. (1996). *Beck Depression Inventory – II Manual*. San Antonio, Texas: The Psychological Corporation.

Becoña, E. (1994). Evaluación de la conducta de fumar. En J. L. Graña (Ed.), *Conductas adictivas: Teoría, evaluación y tratamiento* (pp. 403-454). Madrid: Debate.

Becoña, E. (2004). Tratamiento psicológico del tabaquismo. *Adicciones, 16 (Supl. 2),* 237-263.

Becoña, E. y García, M. P. (1995). Relation between the Tolerance Questionnaire (nicotine dependence) and assessment of carbon monoxide in smokers who participated in treatment for smoking. *Psychological Reports, 77,* 1299-1304.

Becoña, E., López, A., Fernández del Río, E., Míguez, M. C. y Castro, J. (2010). Spanish adaptation of the NDSS (Nicotine Dependence Syndrome Scale) and assessment of nicotine-dependent individuals at Primary Care Health Centers in Spain. *The Spanish Journal of Psychology, 13,* 951-960.

Becoña, E., López-Durán, A., del Río, E. F. y Martínez, Ú. (2014). Changes in the profiles of smokers seeking cessation treatment and in its effectiveness in Galicia (Spain) 2001-10. *BMC Public Health*, *14,* 613.

Becoña, E. y Vázquez, F. L. (1998). The Fagerström Test for Nicotine Dependence in a Spanish sample. *Psychological Reports, 83,* 1455-1458.

Ben-Porath, Y. y Tellegen, A. (2009). *Inventario Multifásico de Personalidad de Minnesota-2 Reestructurado (MMPI-2-RF) (adaptación española realizada por Santamaría Fernández P.).* Madrid: Tea ediciones.

Callaghan, R. C., Veldhuizen, S., Jeysingh, T., Orlan, C., Graham, C., Kakouris, G., … Gatley, J. (2014). Patterns of tobacco-related mortality among individuals diagnosed with schizophrenia, bipolar disorder, or depression. *Journal of Psychiatric Research, 48,* 102-110.

Colom, F., Vieta, E., Martínez-Arán, A., Garcia-Garcia, M., Reinares, M., Torrent, C., ... Salamero, M. (2002). Versión española de una escala de evaluación de la manía: validez y fiabilidad de la Escala de Young. *Medicina Clínica*, *119,* 366-371.

Conway, M., Csank, P. A. R., Holm, S.L. y Blake, C. K. (2000). On assessing individual differences in rumination on sadness. *Journal of Personality Assessment, 75,* 404-425

Cuijpers, P., Van Straten, A. y Warmerdam, L. (2007). Behavioral activation treatments of depression: A meta-analysis. *Clinical Psychology Review*, *27,* 318-326.

Edwards, G. (1986). The alcohol dependence syndrome: A concept as stimulus to enquire. *Addiction, 81,* 171-183. doi: 10.1111/j.1360-0443.1986.tb00313.x

Edwards, G. y Gross, M. M. (1976). Alcohol dependence: Provisional description of a clinical syndrome. *British Medical Journal, 1,* 1058-1061.

Ekers, D., Webster, L., Van Straten, A., Cuijpers, P., Richards, D. y Gilbody, S. (2014). Behavioural activation for depression; an update of meta-analysis of effectiveness and sub group analysis. PLoS ONE 9(6): e100100.

Expósito, F. y Moya, M. (1993). Validación de la UCLA Loneliness Scale en una muestra española. En F. LosCertales y M. Marín (Comps.). *Dimensiones psicosociales de la educación y de la comunicación*, 355-364.

Fagerström, K. O., Kunze, M., Schoberberger, R., Breslau, N., Hughes, J. R., Hurt, R. … Zatonski, W. (1996). Nicotine dependence versus smoking prevalence: Comparison among countries and categories of smokers. *Tobacco Control, 5,* 52-56.

Gierisch, J. M., Bastian, L. A., Calhoun, P. S., McDuffie, J. R. y Williams, J. W. (2012). Smoking cessation interventions for patients with depression: a systematic review and meta-analysis. *Journal of General Internal Medicine, 27,* 351-360.

Goodwin, R. D., Zvolensky, M. J., Keyes, K. M. y Hasin, D. S. (2012). Mental disorders and cigarette use among adults in the United States. *American Journal on Addictions, 21,* 416-423.

Heatherton, T. F., Kozlowski, L. T., Frecker, R. C. y Fagerström, K. O. (1991). The Fagerström Test for Nicotine Dependence: A revision of the Fagerström Tolerance Questionnaire. *British Journal of Addictions, 86*, 1119-1127.

Hervás, G. (2008). Adaptación al castellano de un instrumento para evaluar el estilo rumiativo: la escala de respuestas rumiativas. *Revista de Psicopatología y Salud, 13*, 111-121.

Hopko, D. R., Lejuez, C. W., LePage, J. P., Hopko, S. D. y McNeil, D. W. (2003). A brief behavioral activation treatment for depression. *Behavior Modification, 27,* 458-469.

Hughes, J. R. (2007). Depression during tobacco abstinence. *Nicotine and Tobacco Research, 9,* 443-446.

Instituto Nacional de Estadística (2013). *Encuesta Nacional de Salud: ENSE 2011-2012*. Recuperado de http://www.msc.es/estadEstudios/estadisticas/encuestaNacional/encuestaNac2011/NotaTecnica2011-12.pdf

Kanter, J. W., Mulick, P. S., Busch, A. M., Berlin, K. S., & Martell, C. R. (2007). The Behavioral Activation for Depression Scale (BADS): Psychometric properties and factor structure. *Journal of Psychopathology and Behavioral Assessment*, *29*, 191-202.

Lasser, K., Boyd, W., Woolhandler, S., Himmelstein, D. U., McCormick, D. y Bor, D. (2000). Smoking and mental illness: A population-based prevalence study. *Journal of the American Medical Association, 284,* 2606-2610.

Lejuez, C. W., Hopko, D. R. y Hopko, S. D. (2001). A brief behavioral activation treatment for depression treatment manual. *Behavior Modification*, *25*, 255-286.

Organización Mundial de la Salud (2013). Trastornos Mentales. Depresión. Recomendaciones.http://www.who.int/mental_health/mhgap/evidence/depression/q4/es/

Royal College of Physicians (2013). *Smoking and mental health.* Londres: Royal College of Physicians.

Russell, D. W. (1996). UCLA Loneliness Scale (Version 3): Reliability, validity, and factor structure. *Journal of personality assessment*, *66*(1), 20-40.

Sanz, J., Perdigón, A. L. y Vásquez, C. (2003). Adaptación española del Inventario para la Depresión de Beck-II (BDI-II): 2. Propiedades psicométricas en población general. *Clínica y Salud, 14*, 249–280.

Shaver, P. R. y Brennan, K. A. (1991). Measures of depression and loneliness. *Measures of personality and social psychological attitudes*, *1*, 195-290.

Shiffman, S., Waters, A. J. y Hickcox, M. (2004). The Nicotine Dependence Syndrome Scale: A multidimensional measure of nicotine dependence. *Nicotine & Tobacco Research, 6,* 327-348. doi: 10.1080/14622200400020248l

Shinohara, K., Honyashiki, M., Imai, H., Hunot, V., Caldwell, D., Davies, P., ... Churchill, R. (2013). Behavioural therapies versus other psychological therapies for depression. *Cochrane Database Systematic Review, 10*.

U.S.D.H.H.S. (2014). *The health consequences of smoking-50 years of progress. A report of the Surgeon General*. Rockville, MD: U. S. Department of Health and Human Services, Public Health Services, Office of the Surgeon General.

van der Meer, R. M., Willemsen, M. C., Smit, F. y Cuijpers, P. (2013). Smoking cessation interventions for smokers with current or past depression. *Cochrane Database Systematic Review, 8*.

Weinberger, A. H., Mazure, C. M., Morlett, A. y McKee, S. A. (2013). Two decades of smoking cessation treatment research on smokers with depression: 1990–2010. *Nicotine & Tobacco Research*, *15*, 1014-1031.

World Health Organization (2009). *WHO Tobacco framework convenion on tobacco control.* Ginebra, Suiza.

Young, R. C., Biggs, J. T., Ziegler, V. E. y Meyer, D. A. (1978). A rating scale for mania: reliability, validity and sensitivity. *British Journal of Psychiatry*, *133*, 429-435.

Zvolensky, M. J., Bakhshaie, J., Sheffer, C., Perez, A.y Goodwin, R. D. (2015). Major depressive disorder and smoking relapse among adults in the United States: A 10-year, prospective investigation. *Psychiatry Research*, 226, 73-77.

# ANEXO I

## Consentimiento informado

1. Después de que me han explicado detalladamente el estudio, estoy dispuesto a participar libremente en el mismo, aceptando ser asignado aleatoriamente a uno de los dos grupos de intervención y tratamiento psicológico para dejar de fumar, o a un grupo de espera de tres meses para que luego me apliquen el tratamiento.

2. Con la firma del presente contrato, me comprometo a:

a) Acudir a las 8 sesiones de tratamiento, realizando con el mayor aprovechamiento posible todas las tareas que se recomiendan en cada sesión.

b) Cumplimentar los cuestionarios de evaluación antes del tratamiento, al final del tratamiento y en los seguimientos al mes, tres, seis y doce meses. Los seguimientos se harán personalmente en la Unidad de Tabaquismo y Trastornos Adictivos de la Universidad de Santiago de Compostela. También autorizo a poder hacerlos, de ser necesario, por teléfono o en mi domicilio, a los 6 y 12 meses, así como poder contactar después del año para posteriores seguimientos.

c) Dar el consentimiento para que se puedan grabar las sesiones de evaluación y tratamiento en vídeo para su posible utilización posterior para fines didácticos.

d) Dar el consentimiento para que los datos obtenidos en esta evaluación, en el tratamiento y seguimientos sean utilizados con fines científicos y/o de investigación.

e) Me han informado de que puedo abandonar el Programa para dejar de fumar en cualquier momento, sin dar motivos y sin que esto me suponga ningún perjuicio.

3. Por su parte, el terapeuta que le aplique el tratamiento se compromete a:

a) Aplicar el programa de tratamiento para dejar de fumar al que el participante sea asignado.

b) Mantener la confidencialidad tanto del nombre como de los datos personales obtenidos en las evaluaciones y a lo largo del tratamiento o en cualquier otro aspecto que usted nos indique, de acuerdo a la Ley 41/2002, de 14 de noviembre, básica reguladora de la autonomía del paciente y de derechos y obligaciones en materia de información y documentación clínica.

c) Proporcionarle cualquier información adicional que usted desee sobre del programa de intervención y tratamiento psicológico, evaluación, tareas, etc.

4. Este contrato no es un acuerdo legal, aunque si es un acuerdo al que libremente han llegado ambas partes y que aceptan en su totalidad.

En Santiago de Compostela, a de de 201

Nombre y firma del fumador

*Nombre y firma de la persona que hace la evaluación*
