# Supplementary material for: Cognitive-behavioral treatment with behavioral activation for smoking cessation: Randomized controlled trial
Source: PLoS One. 2019 Apr 8;14(4):e0214252. doi: 10.1371/journal.pone.0214252 (PMC6453447; doi:10.1371/journal.pone.0214252)
Supplement: S3 Text — (PDF) [file pone.0214252.s003.pdf]

SUMMARY OF THE RESEARCH PROJECT

ASSESSMENT OF A PSYCHOLOGICAL TREATMENT FOR SMOKING CESSATION AND MOOD IMPROVEMENT

RESEARCHERS

Elisardo Becoña Iglesias
University of Santiago de Compostela. Faculty of Psychology
Departament of Clinical Psychology and Psychobiology

Carmen Senra Rivera
University of Santiago de Compostela. Faculty of Psychology
Departament of Clinical Psychology and Psychobiology

Mª Carmen Míguez Varela
University of Santiago de Compostela. Faculty of Psychology
Departament of Clinical Psychology and Psychobiology

Carmela Martínez Vispo
University of Santiago de Compostela. Faculty of Psychology
Departament of Clinical Psychology and Psychobiology

Ana López Durán
University of Santiago de Compostela. Faculty of Psychology
Departament of Clinical Psychology and Psychobiology

Elena Fernández del Río
University of Zaragoza
Departament of Psychology and Sociology

Rubén Rodríguez Cano
University of Santiago de Compostela. Faculty of Psychology
Departament of Clinical Psychology and Psychobiology

ABSTRACT

Smoking is the leading preventable cause of morbidity and mortality worldwide. Although there are effective psychological treatments to quit smoking, which have good abstinence results, recently there has been a decrease in quit rates at the end of treatment, and higher rates of relapse.

On the other hand, it is well-known that there is a relationship between depression and smoking. In fact, people with depression are twice as likely to smoke. In the last ten years, in our context we have observed an increase in the number of smokers with history of depression (past or current depression) seeking psychological treatment to quit smoking. For this reason we considered that a more intensive intervention, with components of behavioral activation for depressed mood management, could improve the abstinence outcomes and, therefore, the effectiveness of a cognitive-behavioral smoking cessation intervention.

The aim of the present study is to assess the efficacy of a psychological treatment to quit smoking with specific components of behavioral activation.

We will have three conditions with at least 250 participants, which will be randomly assigned to one of the following conditions. The first condition will be the standard smoking cessation intervention (n = 100), the second condition will be the standard smoking cessation intervention plus behavioral activation components for depressed mood management (n = 100) and the third condition will be a delayed treatment control group (n = 50). The first two conditions will consist of 8 group weekly intervention sessions of 1 hour duration during 8 weeks; and follow-ups at 3, 6 and 12 months after the end of the intervention.

Through this study we expect to know whether the inclusion of specific components for mood management increase abstinence outcomes of a standard psychological smoking cessation treatment, as well as to examine whether the participants experience an improvement in depressive symptoms.

INTRODUCTION

Smoking is the leading preventable cause of morbidity and mortality nowadays in most countries (World Health Organization, 2009; U.S.D.H.H.S., 2014). In Spain, the prevalence of tobacco consumption is one of the highest in Europe (OECD, 2014). The mortality attributed to tobacco consumption is over 50.000 people per year in our country (Banegas et al., 2011; Hernández-García et al. al., 2010). Although in recent years, the prevalence of tobacco smoking in Spain has experienced a significant reduction, reaching the lowest rate in the last 25 years, 24% in 2013 (Instituto Nacional de Estadística, 2013), it still smokes an important percentage of the Spanish population and it continues to be a really important risk factor for health and quality of life.

It is well-know that smoking is related to poor physical and mental health. Epidemiological and clinical studies have found a relationship between smoking and different mental disorders (Becoña, 2004, Goodwin, Zvolensky, Keyes and Hasin, 2012). In fact, tobacco dependence rates are higher in people with mental disorders when comparing to the general population. Besides, consumption and dependence level are associated with mental disorders severity (Royal College of Physicians, 2013).

One of most frequently mental disorders associated with smoking is depression. People with depressive symptoms or with a depressive disorder have a very high prevalence of cigarette smoking (Lasser, Boyd, Woolhandler, Himmelstein, McCormick and Bor, 2000; Tjora, Hetland, Aarø, Wold, Wiium and Øverland, 2014). In addition, they tend to show greater cigarette dependence, greater negative changes in mood due to the withdrawal syndrome, higher rates of relapse after quitting and higher risk of morbidity and mortality attributable to tobacco use (Weinberger, Mazure, Morlett and McKee, 2013; Zvolensky, Bakhshaie, Sheffer, Perez and Goodwin, 2015). In fact, tobacco is responsible for 50% of deaths in patients with depressive disorders (Callaghan et al., 2014).

Nowadays, although we have effective psychological interventions to quit smoking, it has been found that abstinence rates in people with current depression or depressive symptoms are significantly lower than for those without history of major depressive disorder. This could be due to they have a more intense withdrawal syndrome or due to their mood worse when they quit smoking (Gierisch, Bastian, Calhoun, McDuffie and Williams, 2012). Research has suggested that quitting smoking could also increase the likelihood of recurrence of a major depressive disorder in people with past history of depression (Becoña, 2004, Hughes, 2007). Therefore, as some

researchers point out, this type of population may require a more intensive intervention, since they tend to have a greater cigarette dependence, are more likely to suffer negative changes in mood, they are more likely to relapse and have also a higher risk of morbidity and mortality related to tobacco use (Weinberger et al., 2013).

Behavioral activation, which is a behavioral technique, could be defined as a structured, flexible, and brief intervention that focused on making behavioral changes and modifying the environment in order to people reconnect with sources of positive reinforcement (Cuipjers, Van Straten and Warmerdam, 2007). Behavioral activation is based on the idea that the behaviors that characterize depressed people play a significant role in how they feel. This intervention is considered an effective intervention for depression, and has been empirically validated in different studies as a treatment for depression, being considered an effective treatment modality in different clinical samples and diverse contexts (Cuijpers et al., 2007; Ekers, Webster , Van Straten, Cuijpers, Richards and Gilbody, 2014, Hopko, Bell, Armento, Hunt and Lejuez, 2005, MacPherson et al, 2010). In fact, behavioral activation is one of the interventions recommended by the World Health Organization (2013) for depression treatment. Therefore, behavioral activation is an effective treatment for depression, that is relatively simple and time-efficient that may be also suitable for smoking cessation.

In the last ten years, and specifically in our context, there has been an increase in the number of smokers with history of depression (having had in the past or currently having an episode of major depression) seeking treatment to quit smoking (Becoña, López-Durán, del Río and Martínez, 2014). For this reason we consider that a more intensive intervention, with behavioral activation components to manage mood and depressive symptomatology, could improve abstinence outcomes, and therefore, the efficacy of the psychological treatment to quit smoking.

OBJECTIVES

Taking into account that cigarette smoking is the first preventable cause of death, and that quitting smoking is related to experience depressive symptoms, to incorporate new components into smoking cessation treatments could improve abstinence rates and prevent smoking relapse. Through the behavioral activation approach, exposure to positive reinforcing alternatives to smoking cigarettes would be increased, and the distress of the withdrawal syndrome could be reduced, resulting in a possible improvement of smoking abstinence rates and mood.

Therefore, the main aim of this study is to develop and assess a psychological intervention to quit smoking and to manage mood, based on the technique of behavioral activation. We are interested in examine whether the incorporation of behavioral activation is associated with better abstinence outcomes at short and long term, as well as with an improvement in depressive symptomatology.

General objectives:

1. To design and develop a psychological intervention to quit smoking with components from the behavioral activation approach for managing depressed mood.
2. To assess the efficacy of this intervention through a randomized control trial with three groups (standard treatment, standard treatment plus behavioral activation, and control group) in terms of smoking abstinence rates (at the end of treatment and at 3-, 6-, and 12-month follow-ups).
3. To assess whether the applied treatment with components of behavioral activation improves depressed mood compared with a standard smoking cessation treatment, at short, medium and long-term (at the end of treatment and at 3-, 6-, and 12-month follow-ups).

METHOD

Sample

The sample will be composed of seeking treatment smokers at the Smoking Cessation and Addictive Disorders Unit of the University of Santiago de Compostela. Participants will be recruited through the media, posters in healthcare centers, word of mouth, or they will be referred to the unit by their primary care physician or other specialized services of the healthcare system.

The 250 participants meeting the inclusion and exclusion criteria will be randomized to one of the following conditions:

1. Standard cognitive-behavioral smoking cessation treatment (n= 100).
2. Standard cognitive-behavioral smoking cessation treatment plus behavioral activation (n= 100).
3. 3-month delayed treatment control group (n= 50).

The three study conditions will be described in the next pages.

Inclusion/exclusion criteria

Inclusion

- Aged 18 or over.
- Wishing to participate in the treatment program named *"Programa para dejar de fumar"*.
- Smoking 8 or more cigarettes per day before entering in the study.
- Providing written informed consent.

Exclusion

- Diagnosis of a severe mental disorder (bipolar disorder and/or psychotic disorder).
- Concurrent substance use disorder (alcohol, cannabis, stimulant, hallucinogen and/or opioid);
- Having participated in the same or similar treatment over the previous year.
- Having received pharmacological smoking cessation treatment (nicotine replacement therapy, bupropion, or varenicline) over the previous year;
- Presence of a high life-risk pathology that would require immediate individual intervention (i.e., recent myocardial infarction).
- Smoking tobacco products other than cigarettes (i.e., cigars).

Assessment

Two baseline assessment sessions will be carried out in a face-to-face interview, and the following instruments will be administered.

- *Smoking Habit Questionnaire* (Becoña, 1994). It is an instrument consisting of 56 items designed to gather information both on sociodemographic variables (gender, age, marital status, educational level), tobacco use (i.e., number of cigarettes smoked per day), previous attempts to quit or reduce cigarettes, reasons to quit smoking in previous attempts, and beliefs about health related consequences of tobacco.
- Stages of change were assessed with the Stages of Change Questionnaire (Prochaska, Diclemente, & Norcross, 1992). The stages of change model consider addictive behaviors as a continuum in which the person goes through different stages of recovery and relapse until finally achieving abstinence at short, medium and long term. The stages are: precontemplation (the smoker

begins to contemplate smoking as a problem but does not consider to quit smoking at least in the next 6 months), contemplation (the smoker begins to contemplate smoking as a problem, actively seek information and seriously consider the possibility of quitting tobacco in the next 6 months), preparation for action (the smoker has already made the decision to change in the next 30 days), action (the smoker quit smoking and remain abstinent for at least 24 hours), maintenance (6 months or more of abstinence) and finalization (the ex-smoker has not smoked for 5 years or more, having an absence of the desire to smoke cigarettes in any situation and has confidence in remain abstinent).

- *Fagerström Test for Cigarette Dependence (FTCD;* Heatherton, Kozlowski, Frecker y Fagerström, 1991; Becoña, 1994; Becoña y Vázquez, 1998). It is made up of six items for the assessment of cigarette dependence. Scores ≥ 6 are considered to be indicative of dependence (Fagerström et al., 1996). This instrument has been validated with physiological measures of nicotine content in blood, so it can be used to measure the degree of physiological dependence. It has been shown that the relationship between scores in this questionnaire and the physiological measures of smoking is high (Becoña y García, 1995). The FTND is the most widely used instrument for rating nicotine dependence and previous studies have confirmed the reliability of the FTND in different settings and populations.
- *Nicotine Dependence Syndrome Scale (*NDSS; Shiffman, Waters y Hickcox, 2004; Becoña, López, Fernández del Río, Míguez y Castro, 2010). Questionnaire based on multidimensional conceptualization of substance dependence as a syndrome (Edwards, 1986; Edwards y Gross, 1976). It is a self-reported instrument with 19 ítems to assess nicotine dependence.
- *Self-Efficacy/Temptation to Smoke Inventory–Short Form* (Velicer et al., 1990). It consist on 9 items assessing temptation to smoke in different situations: possitive affect situations/social situations; negative affect sitations; and craving/habit.
- *Minnesota Nicotine Withdrawal Scale* (MNWS; Hughes y Hatsukami, 1986). This is an 8-item scale measuring nicotine withdrawal symptoms (depression, insomnia, irritability /frustration /anger, anxiety, difficulty concentrating,

restlessness, increased appetite /weight gain) and craving (desire or urge to smoke, which is considered independently).

- Structured Clinical Interview, according to DSM-5 criteria for assessing tobacco use disorder.
- *Screening Questionnaire Major Depressive Episode* (MDE, Muñoz, 1998). This is an instrument to detect past and current major depressive episodes. It is based on DSM diagnostic criteria for mayor depressive episode.
- *Hamilton Depression Rating Scale* (HRDS; Hamilton, 1960). This instrument was designed to quantitatively assess the severity of depressive symptoms and to monitor changes.
- *Beck Depression Inventory II* (BDI-II; Beck, Steer y Brown, 1996; Sanz, Perdigón and Vásquez; 2003). This is a 21-item self-report scale measuring current depressive symptoms in people of general and clinical population, being 13 and over. This istrument aims to describe the presence and degree of depressive symptoms in these populations, without pretending to establish a clinical diagnosis (Beck et al., 1996). Each item is scored according to a scale of 0 to 3, and the maximum total score that can be obtained is 63.
- *Environmental Reward Observation Scale (*EROS, Armento y Hopko, 2007; Barraca y Pérez-Álvarez, 2010). This is a 10-item brief self-report designed to obtain information on the amount and availability of environmental reward. Higher scores are related to rewarding behaviors and positive affect as a result of reinforcing experiences from the environment.
- *Behavioral Activation for Depression Scale, (*BADS; Kanter, Mulick, Busch, Berlin y Martell, 2007; Barraca, Pérez-Álvarez y Bleda, 2011). This 25-item questionnaire was designed to measure four basic dimensions of the behavioral activation model: Activation, Avoidance/Rumination, Work/School Impairment, and Social Impairment.
- *Los Angeles Loneliness Scale* (UCLA-3; Russell, 1996; Expósito y Moya, 1993). It is self-report measure that consists in 20 items, with four options corresponding to the frequency of the item (never, sometimes, often, and always). This scale has been the most widely used in the studies on loneliness and has good reliability indices (Shaver and Brennan, 1991). The scores obtained have been related to social behavior, attribution patterns and feelings of

abandonment, depression, emptiness, helplessness, isolation, among others. The higher the score obtained in the scale, the higher the level of loneliness, with scores ranging between 20 and 80.

- *Ruminative Response Scale* (RRS, Nolen-Hoeksema, Larson y Grayson, 1999; Hervás, 2008). It is a 22-item self-report for assessing ruminative coping responses to depressed mood. In 2003, Treynor and colleagues found that 12 items from the RRS overlapped with depressive symptoms, so that the resulting 10-item version will be used These 10 items supported a two-factor model: Brooding (5 items) and Reflection (5 items). The Spanish translation of the RRS has been found to have adequate psychometric properties.
- *Smoking self-reports*. These are sheets that are provided to participants in order to know in depth their smoking behavior. On each sheet, the participant must cover daily the following sections: number of cigarettes (up to 40 cigarettes per sheet), time at which they smoke each cigarette, cigarette pleasure (from 0 to 10, being 0 the minimum pleasure and 10 the maximum) and finally, the situation in which the participant smoke.
- *Carbon monoxide in expired air assessment (CO).* We will use the Micro+ Smokerlyzer (Bedfont Scientific Ltd, Maidstone, Kent, U.K.) to measure carbon monoxide (CO) in expired air so as to corroborate self-reported abstinence at the end of treatment and at follow-ups, as suggested in previous studies. This device provides a digital reading of the particles per million (ppm) of CO and can be transformed into the equivalent percentage of COHb (carboxyhemoglobin) in blood.
- *End of treatment questionnaire*. In the last treatment session (session 8), participants will complete the end-of-treatment assessment questionnaire (Becoña and Míguez, 1995) in which the following variables will be evaluated: current consumption of cigarettes (number of cigarettes smoked the present day and the previous one and brand consumed); estimated date to quit smoking (if the participant has not quit smoking during the treatment period); social support received from the people in your environment; physical and psychological improvements experienced since the beginning of the smoking cessation intervention; possible worsening during the weeks of treatment; the tobacco withdrawal symptom scale from Hughes and Hatsukami (1986); assessment of

confidence in remaining abstinent in the next 6 months on a scale of 0 (not at all) to 10 (maximum); assessment of satisfaction with the services received (excellent, good, fair or poor) through the Client Satisfaction Questionnaire (CSQ-8) of Larsen, Attkinson, Hargreaves and Nguyen (1979); comments about the intervention procedures and the smoking cessation process.

- *Follow-up questionnaires*. Self-report instrument collecting data about abstinence and / or relapse at 3, 6 and 12 months of follow-up.

Procedure

The 250 participants will be randomized according to a computer generated allocation sequence (ratio: 2.2.1.): (1) standard cognitive-behavioral smoking cessation treatment; (2) standard cognitive-behavioral smoking cessation treatment plus behavioral activation; or (3) a 3-month delayed treatment control group.

After condition assignment, both active treatments will be administered in eight weekly 60-minute sessions. At the end of treatment, there will be a post-treatment assessment (during session 8) and face-to-face follow-ups at 3, 6, and 12 months.

Participants in the wait list control group will be assessed after the 3 months of waiting. After that, participant will be offered to participate in a smoking cessation treatment.

Description of study conditions

Treatment conditions will be exclusively cognitive-behavioral-based interventions. A full session-by-session treatment components description is provided in Tables 1 and 2.

Standard cognitive-behavioral smoking cessation treatment

The standard cognitive-behavioral smoking cessation treatment is a manualized treatment for tobacco dependence, named “Smoking Cessation Program”. The treatment components are: treatment contract, self-report and graphic representation of cigarette consumption, information about tobacco, nicotine fading (change of cigarette brands each week, progressively decreasing the intake of nicotine and tar), stimulus control, activities to prevent withdrawal syndrome, physiological feedback (CO in expired air) on cigarette consumption, and relapse-prevention strategies (assertion training, problem-solving training, changing tobacco-related misconceptions, management of anxiety and

anger, exercise, weight control, and self-reinforcing). Treatment will be delivered in eight 60-minute sessions over 8 consecutive weeks.

Table 1

Summary of session-by-session intervention procedures for the Cognitive-behavioral Smoking Cessation Treatment (CBSCT)

Session	Components
1	Overview of treatment<br>Smoking cessation treatment rationale<br>Review self-monitoring (tracking cigarettes and smoking antecedents and consequences)<br>Indications about how to graphically represent the No. of cigarettes/day<br>Reasons for smoking and for quitting<br>Discuss smoking history and quit experiences<br>Explain and provide written materials about tobacco, nicotine dependence, smoking health consequences and quit smoking benefits<br>Explain nicotine fading through brand change<br>Physiological feedback (CO)<br>As homework:<br>- Brand change<br>- Communicate to at least one person [family, friend, coworker, etc.) that he/she is trying to quit smoking in the next 30 days<br>- Not smoke more cigarettes than the average of those smoked the previous week<br>- Leave a third of the cigarette without smoking<br>- Refuse cigarette offers
2	Check homework and nicotine fading compliance<br>Continue smoking self-monitoring and analyze smoking behavior during the week<br>Physiological feedback (CO)<br>Discuss brand change difficulties<br>New brand change and reduce No. of cigarettes<br>Review importance of social support

	Introduce stimulus control technique to remove situations conditioned to smoking<br>Nicotine withdrawal and strategies to avoid it<br>Breathing exercises and relaxation techniques (practice as homework)
3	Check nicotine fading, cigarette reduction and stimulus control compliance<br>Check breathing exercises compliance and strategies to avoid withdrawal symptoms<br>Physiological feedback (CO)<br>Continue cigarette self-monitoring and analyze smoking behavior<br>New brand change and reduce No. of cigarettes<br>Continue stimulus control technique<br>Explain and provide written materials for weight control and exercise<br>Continue with breathing exercises and strategies to avoid withdrawal symptoms
4	Check nicotine fading, cigarette reduction and stimulus control compliance<br>Check breathing exercises compliance and strategies to avoid withdrawal symptoms<br>Physiological feedback (CO)<br>Continue smoking self-monitoring and analyze smoking behavior<br>New brand change and reduction of No. of cigarettes<br>Continue stimulus control technique<br>Stress and anxiety management strategies<br>Problem-solving training
5	Check nicotine fading, cigarette reduction, and stimulus control compliance<br>Physiological feedback (CO)<br>Continue smoking self-monitoring and analyze smoking behavior<br>New brand change and reduction of No. of cigarettes<br>Management of anxiety and anger<br>Self-reinforcing<br>Changing tobacco-related misconceptions
6	Quitting experience and withdrawal symptoms<br>Physiological feedback (CO)<br>Discuss and plan for high-risk lapse and relapse situations

	Motivating factors for maintaining abstinence<br>Benefits of quitting smoking<br>Common barriers for maintaining abstinence
7	Quitting experience and withdrawal symptoms<br>Physiological feedback (CO)<br>Discuss and plan for high-risk lapse and relapse situations<br>Motivating factors for maintaining abstinence<br>Benefits of quitting smoking<br>Strategies for relapse prevention
8	Physiological feedback (CO)<br>Managing the future as ex-smokers<br>Encouragement for abstinence maintenance<br>Support for lapses and relapse<br>Review motivating factors, lifestyle changes, physical and cognitive-behavioral health improvement<br>Treatment conclusion and management of potential obstacles

Standard cognitive-behavioral smoking cessation treatment with behavioral activation

Behavioral Activation will be applied along with the previously described standard cognitive-behavioral smoking cessation treatment. The treatment elements are the above-mentioned ones plus the following: analysis of the relationship between behavior and mood, identification of situations and behaviors that decrease mood, identifying avoidance behaviors, and identifying rumination and worry, self-report of pleasant daily activities, pleasant activity scheduling to increase engagement in rewarding activities and to reduce patterns of behavioral avoidance. Treatment will be delivered in eight 60-minute sessions over 8 consecutive weeks.

Table 2

Summary of session-by-session intervention procedures for the Cognitive-behavioral Smoking Cessation Treatment plus behavioral activation (CBSCT + BA)

Session	Components
1	Overview of treatment

	Smoking cessation treatment rationale<br>Review self-monitoring (tracking cigarettes and smoking antecedents and consequences)<br>Indications about how to graphically represent the No. of cigarettes/day<br>Reasons for smoking and for quitting<br>Discuss smoking history and quit experiences<br>Explain and provide written materials about tobacco, nicotine dependence, smoking health consequences and quit smoking benefits<br>Explain nicotine fading through brand change<br>Physiological feedback (CO)<br>As homework:<br>- Brand change<br>- Communicate to at least one person (family, friend, coworker, etc.) that he/she is trying to quit smoking in the next 30 days<br>- Not smoke more cigarettes than the average of those smoked the previous week<br>- Leave a third of the cigarette without smoking<br>- Refuse cigarette offers
2	Check homework and nicotine fading compliance<br>Continue smoking self-monitoring and analyze smoking behavior during the week<br>Physiological feedback (CO)<br>Discuss brand change difficulties<br>New brand change and reduce No. of cigarettes<br>Review importance of social support<br>Introduce stimulus control technique to remove situations conditioned to smoking<br>Nicotine withdrawal and strategies to avoid it<br>Breathing exercises and relaxation techniques (practice as homework)
3	Check nicotine fading, cigarette reduction and stimulus control compliance<br>Check breathing exercises compliance and strategies to avoid withdrawal symptoms<br>Physiological feedback (CO)

	Continue cigarette self-monitoring and analyze smoking behavior<br>New brand change and reduce No. of cigarettes<br>Continue stimulus control technique<br>Explain and provide written materials for weight control and exercise<br>Continue with breathing exercises compliance and strategies to avoid withdrawal symptoms<br>Rationale of mood influence in smoking cessation (provide written materials)<br>Homework: daily activities self-monitoring
4	Check nicotine fading, cigarette reduction and stimulus control compliance<br>Check breathing exercises compliance and strategies to avoid withdrawal symptoms<br>Physiological feedback (CO)<br>Continue smoking self-monitoring and analyze smoking behavior<br>New brand change and reduction of No. of cigarettes<br>Continue stimulus control technique<br>Stress and anxiety management strategies<br>Check activity scheduling and encourage recognizing patterns of depressed behavior and the way in which engaging in enjoyable and important activities may impact their overall mood<br>Homework: continue activity scheduling, create a pleasant activities list and choose one to do during the week
5	Check nicotine fading, cigarette reduction, and stimulus control compliance<br>Check activity scheduling, pleasant activities list elaboration, and pleasant activity compliance<br>Physiological feedback (CO)<br>Continue smoking self-monitoring and analyze smoking behavior<br>New brand change and reduction of No. of cigarettes<br>Management of anxiety and anger<br>Self-reinforcing<br>Changing tobacco-related misconceptions<br>Problem-solving training<br>Recognize avoidance behavior and impact on mood<br>Activity scheduling and engagement in 2 pleasant activities/week

6	Quitting experience and withdrawal symptoms<br>Physiological feedback (CO)<br>Discuss and plan for high-risk lapse and relapse situations<br>Motivating factors for maintaining abstinence<br>Benefits of quitting smoking<br>Common barriers for maintaining abstinence<br>Ruminative thoughts, smoking cessation process, and relapse<br>Check activity scheduling and pleasant activity compliance<br>Activity scheduling for the next week and engagement in 2 pleasant activities/week
7	Quitting experience and withdrawal symptoms<br>Physiological feedback (CO)<br>Discuss and plan for high-risk lapse and relapse situations<br>Motivating factors for maintaining abstinence<br>Benefits of quitting smoking<br>Review how behavioral activation impacts their overall mood<br>Review avoidance behavior and ruminative thoughts' significance<br>Strategies for relapse prevention
8	Physiological feedback (CO)<br>Managing the future as ex-smokers<br>Encouragement for abstinence maintenance<br>Support for lapses and relapse<br>Review motivating factors, lifestyle changes, physical and cognitive-behavioral health improvement<br>Review BA strategies<br>Treatment conclusion and management of potential obstacles

Wait list

It will be a delayed-treatment control group for a period of 3 months. After the 3-month period, another assessment will be carried out, and then participants will be offered to participate in a smoking cessation treatment.

Intervention procedures

- Two baseline assessment sessions.

- In both groups 8 sessions will be applied, at a rate of 1 weekly session of 60 minutes, over 8 weeks.
- The psychological intervention will be carried out by a clinical psychologist or general health psychologist. These professionals will receive specific training of each treatments to apply and will be blind to the other treatment.
- An assessment will be carried out at the end of the treatment (session 8) examining relevant variables for the study.
- Follow-ups will be carried out face-to-face, and their corresponding assessments, at 3, 6 and 12 months after the intervention has been completed.
- The abstinence assessment will be done in all cases self-reported and biochemically validated through carbon monoxide in exhaled air, using the Micro + Smokerlizer. Participants will be considered abstinent if they reported no smoking, not even a puff of a cigarette, for ≥ 24 hours at the end of treatmen and had an expired CO reading of ≤ 10 parts per million (ppm). Regarding follow-ups, both point-prevalence-abstinence criterion (Velicer et al., 1992) and the continuous abstinence criterion will be used (West et al., 2005). A participant will be considered abstinent if report abstinence, not even a puff of a cigarette, ≥ 7 days prior to follow-up day at the 3-month follow-up, and had an expired CO reading of ≤ 10 parts per million (ppm). At the 6- and 12-month follow-ups, participants will be considered abstinent if they reported abstinence, not even a puff of a cigarette, for ≥ 30 days prior to follow-up day, and had an expired CO reading of ≤ 10 ppm. In those cases in which it was not possible to locate the participants, they were considered as smokers in the corresponding follow-up.

DATA ANALYSIS

SPSS will be used for statistical analysis. All data analysis will follow the intention-to-treat principle. We will conduct descriptive analysis to summarize the characteristics of the total sample, and the characteristics of the participants in each of the three groups. The main analysis will be a comparison between both active groups and the control group of the proportions of abstinent smokers at the end of treatment, and between BA intervention group and standard intervention group at the end of treatment, and at 3-, 6-, and 12-month follow-ups, through chi-square tests and odds ratios with 95% confidence intervals. *t*-test, regression analysis, repeated measures ANOVA, and mediation analysis will be also conducted.

EXPECTED FINDINGS

- Design and develop an effective psychological treatment to quit smoking that includes elements for the management of depressive symptoms through the technique of behavioral activation.
- Analyze short term efficacy (end of treatment) and long term efficacy (3, 6 and 12 months follow-ups), through a randomized study with three groups: a) standard treatment (smoking cessation intervention), b) standard treatment plus behavioral activation, and c) wait list control group (three months delayed treatment).
- Assess whether the behavioral activation approach intervention to quit smoking, also improves depressive symptomatology in the short (end of treatment) and long term (3, 6 and 12 months follow-us).

Specific expected findings are the following:

1. The two active treatments will obtain better results regarding abstinence rates comparing to the control condition at the end of the treatment.
2. The behavioral intervention approach intervention to quit smoking will be more effective in terms of the abstinence rates than the standard treatment, at the end of treatment and at 3, 6 and 12 months follow-ups.
3. The two active treatments will achieve significant reductions in the scales assessing depressive symptomatology (Beck Depression Inventory II, Hamilton Depression Scale) comparing to the wait list control condition, at the end of treatment.
4. Participants receiving the behavioral intervention approach intervention to quit smoking will achieve significant reductions in the scales assessing depressive symptomatology (Beck Depression Inventory II, Hamilton Depression Scale) comparing to the participants in the standard treatment, at the end of treatment and at 3, 6 and 12 months follow-ups.
5. The two active treatments will achieve a significant improvement in relation to the wait list control group in the variables of craving, self-efficacy and withdrawal syndrome, at the end of treatment.
6. Participants receiving the behavioral intervention approach intervention to quit smoking will have a significant improvement in relation to the standard group in the variables of craving, self-efficacy and withdrawal syndrome, at the end of treatment and at 3, 6 and 12 months follow-ups.

7. The two active treatments will achieve a significant improvement comparing to the wait list control group in the scales assessing behavioral activation-related variables (Observational Scale of Environmental Reinforcement, EROS, Behavioral Activation Scale for Depression, BADS), at the end of treatment.
8. Participants receiving the behavioral intervention approach intervention to quit smoking will have a significant improvement in relation to the standard treatment group in the scales assessing behavioral activation-related variables (Observational Scale of Environmental Reinforcement, EROS, Behavioral Activation Scale for Depression, BADS), at the end of treatment and at 3, 6 and 12 months follow-ups.

REFERENCES

Armento, M. E.y Hopko, D. R. (2007). The environmental reward observation scale (EROS): development, validity, and reliability. *Behavior Therapy, 38*, 107-119.

Barraca, J., Pérez-Álvarez, M., & Bleda, J. H. L. (2011). Avoidance and activation as keys to depression: Adaptation of the Behavioral Activation for Depression Scale in a Spanish sample. *Spanish Journal of Psychology, 14*, 998-1009.

Barraca, J. y Pérez-Álvarez, M. (2010). Spanish adaptation of the Environmental Reward Observation Scale (EROS), *Anxiety and Stress, 16*, 95-107.

Banegas, J. R., Díez-Gañán, L., Bañuelos-Marco, B., González-Enríquez, J., Villar-Álvarez, F., Martín-Moreno, J. M., … Jiménez-Ruíz, C. (2011). Mortalidad atribuíble al consumo de tabaco en España en 2006. *Medicina Clínica, 136,* 97-102.

Banegas, J. R., Díez, L., González, J., Villar, F. y Rodríguez-Artalejo, F. (2005). La mortalidad atribuible al tabaquismo comienza a descender en España. *Medicina Clínica, 124,* 769-771.

Beck, A. T., Steer, R. y Brown, G. (1996). *Beck Depression Inventory – II Manual*. San Antonio, Texas: The Psychological Corporation.

Becoña, E. (1994). Evaluación de la conducta de fumar. En J. L. Graña (Ed.), *Conductas adictivas: Teoría, evaluación y tratamiento* (pp. 403-454). Madrid: Debate.

Becoña, E. (2004). Tratamiento psicológico del tabaquismo. *Adicciones, 16 (Supl. 2),* 237-263.

Becoña, E. y García, M. P. (1995). Relation between the Tolerance Questionnaire (nicotine dependence) and assessment of carbon monoxide in smokers who participated in treatment for smoking. *Psychological Reports, 77,* 1299-1304.

Becoña, E., López, A., Fernández del Río, E., Míguez, M. C. y Castro, J. (2010). Spanish adaptation of the NDSS (Nicotine Dependence Syndrome Scale) and assessment of nicotine-dependent individuals at Primary Care Health Centers in Spain. *The Spanish Journal of Psychology, 13,* 951-960.

Becoña, E., López-Durán, A., del Río, E. F. y Martínez, Ú. (2014). Changes in the profiles of smokers seeking cessation treatment and in its effectiveness in Galicia (Spain) 2001-10. *BMC Public Health, 14,* 613.

Becoña, E. y Vázquez, F. L. (1998). The Fagerström Test for Nicotine Dependence in a Spanish sample. *Psychological Reports, 83,* 1455-1458.

Ben-Porath, Y. y Tellegen, A. (2009). *Inventario Multifásico de Personalidad de Minnesota-2 Reestructurado (MMPI-2-RF) (adaptación española realizada por Santamaría Fernández P.).* Madrid: Tea ediciones.

Callaghan, R. C., Veldhuizen, S., Jeysingh, T., Orlan, C., Graham, C., Kakouris, G., … Gatley, J. (2014). Patterns of tobacco-related mortality among individuals diagnosed with schizophrenia, bipolar disorder, or depression. *Journal of Psychiatric Research, 48,* 102-110.

Colom, F., Vieta, E., Martínez-Arán, A., Garcia-Garcia, M., Reinares, M., Torrent, C., ... Salamero, M. (2002). Versión española de una escala de evaluación de la manía: validez y fiabilidad de la Escala de Young. *Medicina Clínica*, *119,* 366-371.

Conway, M., Csank, P. A. R., Holm, S.L. y Blake, C. K. (2000). On assessing individual differences in rumination on sadness. *Journal of Personality Assessment, 75,* 404-425

Cuijpers, P., Van Straten, A. y Warmerdam, L. (2007). Behavioral activation treatments of depression: A meta-analysis. *Clinical Psychology Review*, *27,* 318-326.

Edwards, G. (1986). The alcohol dependence syndrome: A concept as stimulus to enquire. *Addiction, 81,* 171-183. doi: 10.1111/j.1360-0443.1986.tb00313.x

Edwards, G. y Gross, M. M. (1976). Alcohol dependence: Provisional description of a clinical syndrome. *British Medical Journal, 1,* 1058-1061.

Ekers, D., Webster, L., Van Straten, A., Cuijpers, P., Richards, D. y Gilbody, S. (2014). Behavioural activation for depression; an update of meta-analysis of effectiveness and sub group analysis. PLoS ONE 9(6): e100100.

Expósito, F. y Moya, M. (1993). Validación de la UCLA Loneliness Scale en una muestra española. En F. LosCertales y M. Marín (Comps.). *Dimensiones psicosociales de la educación y de la comunicación*, 355-364.

Fagerström, K. O., Kunze, M., Schoberberger, R., Breslau, N., Hughes, J. R., Hurt, R. … Zatonski, W. (1996). Nicotine dependence versus smoking prevalence: Comparison among countries and categories of smokers. *Tobacco Control, 5,* 52-56.

Gierisch, J. M., Bastian, L. A., Calhoun, P. S., McDuffie, J. R. y Williams, J. W. (2012). Smoking cessation interventions for patients with depression: a systematic review and meta-analysis. *Journal of General Internal Medicine, 27,* 351-360.

Goodwin, R. D., Zvolensky, M. J., Keyes, K. M. y Hasin, D. S. (2012). Mental disorders and cigarette use among adults in the United States. *American Journal on Addictions, 21,* 416-423.

Heatherton, T. F., Kozlowski, L. T., Frecker, R. C. y Fagerström, K. O. (1991). The Fagerström Test for Nicotine Dependence: A revision of the Fagerström Tolerance Questionnaire. *British Journal of Addictions, 86*, 1119-1127.

Hervás, G. (2008). Adaptación al castellano de un instrumento para evaluar el estilo rumiativo: la escala de respuestas rumiativas. *Revista de Psicopatología y Salud, 13*, 111-121.

Hopko, D. R., Lejuez, C. W., LePage, J. P., Hopko, S. D. y McNeil, D. W. (2003). A brief behavioral activation treatment for depression. *Behavior Modification*, *27*, 458-469.

Hughes, J. R. (2007). Depression during tobacco abstinence. *Nicotine and Tobacco Research, 9,* 443-446.

Instituto Nacional de Estadística (2013). *Encuesta Nacional de Salud: ENSE 2011-2012*. Recuperado de http://www.msc.es/estadEstudios/estadisticas/encuestaNacional/encuestaNac2011/NotaTecnica2011-12.pdf

Kanter, J. W., Mulick, P. S., Busch, A. M., Berlin, K. S., & Martell, C. R. (2007). The Behavioral Activation for Depression Scale (BADS): Psychometric properties and factor structure. *Journal of Psychopathology and Behavioral Assessment*, *29*, 191-202.

Lasser, K., Boyd, W., Woolhandler, S., Himmelstein, D. U., McCormick, D. y Bor, D. (2000). Smoking and mental illness: A population-based prevalence study. *Journal of the American Medical Association, 284,* 2606-2610.

Lejuez, C. W., Hopko, D. R. y Hopko, S. D. (2001). A brief behavioral activation treatment for depression treatment manual. *Behavior Modification*, *25*, 255-286.

Organización Mundial de la Salud (2013). Trastornos Mentales. Depresión. Recomendaciones.http://www.who.int/mental_health/mhgap/evidence/depression/q4/es/

Royal College of Physicians (2013). *Smoking and mental health.* Londres: Royal College of Physicians.

Russell, D. W. (1996). UCLA Loneliness Scale (Version 3): Reliability, validity, and factor structure. *Journal of personality assessment*, *66*(1), 20-40.

Sanz, J., Perdigón, A. L. y Vásquez, C. (2003). Adaptación española del Inventario para la Depresión de Beck-II (BDI-II): 2. Propiedades psicométricas en población general. *Clínica y Salud, 14*, 249–280.

Shaver, P. R. y Brennan, K. A. (1991). Measures of depression and loneliness. *Measures of personality and social psychological attitudes*, *1*, 195-290.

Shiffman, S., Waters, A. J. y Hickcox, M. (2004). The Nicotine Dependence Syndrome Scale: A multidimensional measure of nicotine dependence. *Nicotine & Tobacco Research, 6,* 327-348. doi: 10.1080/146222004000202481

Shinohara, K., Honyashiki, M., Imai, H., Hunot, V., Caldwell, D., Davies, P., ... Churchill, R. (2013). Behavioural therapies versus other psychological therapies for depression. *Cochrane Database Systematic Review, 10*.

U.S.D.H.H.S. (2014). *The health consequences of smoking-50 years of progress. A report of the Surgeon General*. Rockville, MD: U. S. Department of Health and Human Services, Public Health Services, Office of the Surgeon General.

van der Meer, R. M., Willemsen, M. C., Smit, F. y Cuijpers, P. (2013). Smoking cessation interventions for smokers with current or past depression. *Cochrane Database Systematic Review, 8*.

Weinberger, A. H., Mazure, C. M., Morlett, A. y McKee, S. A. (2013). Two decades of smoking cessation treatment research on smokers with depression: 1990–2010. *Nicotine & Tobacco Research*, *15*, 1014-1031.

World Health Organization (2009). *WHO Tobacco framework convenion on tobacco control.* Ginebra, Suiza.

Young, R. C., Biggs, J. T., Ziegler, V. E. y Meyer, D. A. (1978). A rating scale for mania: reliability, validity and sensitivity. *British Journal of Psychiatry*, *133*, 429-435.

Zvolensky, M. J., Bakhshaie, J., Sheffer, C., Perez, A.y Goodwin, R. D. (2015). Major depressive disorder and smoking relapse among adults in the United States: A 10-year, prospective investigation. *Psychiatry Research*, 226, 73-77.

SUPPLEMENT MATERIAL

Informed Consent

1. After researchers have explained in detail the study procedures, I am agreeing to participate in it. I agree to be randomly assigned to one of the two intervention groups to quit smoking or to the waiting list group of three months, and then have access to the smoking cessation treatment.
2. By signing this consent, I commit to:
    a) Attend the 8 sessions of treatment, performing with the greatest possible use all the tasks recommended in each session.
    b) Complete the assessment questionnaires before treatment, at the end of treatment and at three, six and twelve month follow-ups. The follow-ups will be made in person at the Smoking Cessation and Addictive Disorders Unit of the University of Santiago de Compostela. I also authorize to do the follow-ups, if necessary, by phone or at home, at 6 and 12 months, as well as to contact with me after the year for subsequent follow-ups.
    c) Consent video recordings of assessments and treatment sessions for possible later use for teaching and researching purposes.
    d) Consent to use the data obtained in the assessment sessions, during the treatment sessions and at follow-ups, for scientific and / or research purposes.
3. I have been informed that I am free to withdraw at any time and without giving a reason. Withdrawing from this study will not cause any problem or inconvenience.
4. Therapists who applies the treatment and researchers agree to:
    a) Apply the smoking cessation treatment to which the participant will be randomized.
    b) Maintain the confidentiality of both the name and personal data obtained in the assessment sessions and throughout the treatment sessions or of any other aspect that the participant indicate, according to Law 41/2002, of November 14, about the autonomy of the patient and rights and obligations in terms of personal and clinical information.
    c) Provide any additional information that the participant want about the intervention, and the assessment sessions.
5. This is not a legal contract, and I voluntarily agree to take part in this study.

Santiago de Compostela, of 201

Name and signature of the participant *Name and signature of researcher taking the consent*
